# Biogenic Waste from Two Varieties of Plantain in Ghana Contain Pectin with Potential Binding Properties in Conventional Tablets

**DOI:** 10.1155/2024/5461358

**Published:** 2024-06-17

**Authors:** Desmond Asamoah Bruce Otu, Frederick William Akuffo Owusu, Mariam El Boakye-Gyasi, Raphael Johnson, Prince George Jnr Acquah, Yayra Edzor-Agbo, Marcel Tunkumgnen Bayor, Mary-Ann Archer

**Affiliations:** ^1^Department of Pharmaceutics, Faculty of Pharmacy and Pharmaceutical Sciences, Kwame Nkrumah University of Science and Technology, Kumasi, Ghana; ^2^Department of Pharmaceutics, School of Pharmacy and Pharmaceutical Sciences, University of Cape Coast, Cape Coast, Ghana

## Abstract

Pharmaceutical formulations have traditionally relied on plants and their derivatives for various APIs and excipients. In Ghana, the widespread utilization of plantains, irrespective of their ripeness, generates significant waste at every stage of processing, posing disposal issues. Fascinatingly, these wastes, often discarded, possess significant economic potential and can be recycled into valuable raw materials or products. Pectin, a polysaccharide that occurs naturally, has seen a surge in interest in recent times. It has found widespread use in the pharmaceutical sector, particularly as a binding agent in tablet formulations. This study aimed to evaluate pectin from two popular plantain varieties, Apem (M) and Apantu (T) at different ripening stages, for pharmaceutical use as a binding agent in immediate-release tablets. The ripening stages selected were the matured-green (G), half-ripe (H), and full-ripe (R). Acid (D) and alkaline (L) mediums of extraction were employed for each ripening stage for both varieties. Wet granulation method was used to prepare the granules using paracetamol as a model drug, and their flow properties were subsequently assessed. Postcompression tests including, hardness, friability, weight uniformity, disintegration, assay, and in vitro dissolution were also assessed. Granules from all formulation batches had good flow properties indicated by their angle of repose (14.93 ± 1.41–21.80 ± 1.41), Hausner ratio (0.96 ± 0.27–1.22 ± 0.02), and compressibility (%) (7.69 ± 0.002–20.51 ± 0.002). All the tablets passed the uniformity of weight with none deviating by ±5%. The hardness of all the formulated tablets ranged between 3.96 ± 0.32 and 13.21 ± 0.36, while the friability for all tablets was below 1%. The drug content was between 100.1 ± 0.23% and 103.4 ± 0.01%. Tablets formulated with pectin as a binding agent at concentrations of 10% w/v and 15% w/v successfully met the disintegration test criteria for immediate release tablets. However, those prepared with a concentration of 20% w/v (MGL, MHD, MHL, MRD, MRL, TGL, THD, THL, and TRL) did not pass the disintegration test. Consequently, all batches of tablets successfully met the dissolution test requirement (Diss, *Q* > 75%), except for the batches that did not pass the disintegration test (Diss, *Q* < 75%). Ultimately, pectins extracted from the peels of Apem and Apantu at different ripening stages using acid and alkaline extraction can be commercially exploited as pharmaceutical binders at varying concentrations in immediate-release tablets.

## 1. Introduction

The active pharmaceutical ingredient, or API, and the excipient are the two basic components of a drug. Without the use of excipients, few medicines, if any, could be produced [1]. Lately, there has been a notable surge in the pursuit of alternative excipients derived from renewable natural sources. Plants and their derivatives have long been used to make a variety of medications and excipients for pharmaceutical formulations [2]. Plants can provide a consistent supply of raw materials because they are renewable and can be grown or harvested sustainably. Lately, food industry waste, comprising both edible and inedible portions, has been harnessed as a raw material to obtain pharmaceutical excipients [3].

In recent years, pectin, a naturally occurring biopolymer, has been extracted from food waste including watermelon rinds, carrots, and okra and found use in the pharmaceutical industry [4, 5]. Due to its low production cost and biodegradability, pectin stands out among the most commercialized biopolymers [6]. Pectin extracted from *Abelmoschus esculentus* L. has been used as a pharmaceutical binder in immediate-release tablets [7].

Pharmaceutical binders are one of the important ingredients added to a powder mix to form granules or tablets [8, 9]. They play an essential role in the granulation process, improving the cohesion and plasticity of the powder mix [10]. This enhances the processability of the tablet and reduces the risk of tablet deformation during manufacture [11].

In West Africa, Ghana is the primary producer of plantain, boasting a substantial output of approximately 3.9 million in tonnage in the year 2016. This noteworthy contribution constituted around 13.12% of the Gross Domestic Product (GDP) in terms of agriculture within the country [12]. Plantain is a nonseasonal crop; hence, it is always in production. Therefore, yield losses resulting from postharvest handling and disease are particularly substantial and range from 20 to 50 percent [12]. It is also noteworthy that, plantain is used in several ways attributable to the ripening stage. Hence, biogenic waste generated in the form of peels from its use at all the ripening stages could be remediated as a potential waste to wealth [13, 14].

The physicochemical properties of plantain peel pectin (PPP) extracted using acid and alkaline mediums at different ripening stages have been reported by Otu et al. [13]. From the results discussed, PPP has the potential to be used in the food and beverage industries as well as the pharmaceutical industries. The potential applications are varied and hence the need for further research to evaluate their potential as a pharmaceutical excipient and probable substitute for commercial pectin.

This study aims to explore the binding qualities of pectin extracted from the Apem and Apantu plantain varieties at different ripening stages as a potential source of pharmaceutical binder for immediate-release tablets. Presently, based on the available information, this study represents the initial investigation documenting the potential use of plantain peel pectin as a binding agent in conventional tablets.

## 2. Materials and Methods

### 2.1. Materials

Plantain peel pectin, paracetamol powder (Ernest Chemist Ltd, Ghana), Tragacanth gum powder BP (Sigma-Aldrich, USA), Corn starch BP (Jilin COFCO Bio-Chem, China) Talc, and lactose BP (Xiʼan Rongsheng Biotechnology, China). All other reagents were of analytical grade and were used as received.

### 2.2. Methods

#### 2.2.1. Preparation of Paracetamol Granules

The wet granulation technique, incorporating the method of geometric dilution, was used to prepare paracetamol granules. Pectin, extracted from plantain peels of Apem (M) and Apantu (T) varieties at different ripening stages, matured-green (G), half-ripe (H), and full-ripe (R), using both acid (D) and alkaline (L) methods, acted as a pharmaceutical binder. This binder was added to the formulation in varying concentrations. The process included screening the damp mass through a 2.36 mm mesh, drying the granules in a hot air oven at 60°C for 1 hour, and screening the dry granules through a 1.18 mm mesh. Talc was incorporated as an extragranular lubricant. The formulations were prepared with different binder concentrations; *F*1–*F*6 and *F*7–*F*12 at 10%, *F*14–*F*19 and *F*20–*F*25 at 15%, and *F*27–*F*32 and *F*33–*F*38 at 20%. These concentrations were based on the weight/volume of the respective pectin types MGD, MGL, MHD, MHL, MRD, and MRL and TGD, TGL, THD, THL, TRD, and TRL. Tragacanth BP, the standard binder, was used in formulations *F*13, *F*26, and *F*39 at concentrations of 10%, 15%, and 20%, respectively. Each tablet was compressed from 650 mg of granules using a Cadmach CTX 26 tableting machine (Cadmach Machinery Co. Pvt. Ltd., India) at pressures of 45–50 kN. The composition for the formulation of the granules is described in Table 1. The quantities were increased in order to generate granules of sufficient quantity for the formulation of one hundred (100) tablets. Formulated granules are shown in Supplementary file (available ), “PPP Binder granules.”

#### 2.2.2. Evaluation of the Granules' Flow Properties for the Various Batches

A quantity of 20 grams of granules was transferred into a measuring cylinder (100 mL) and gently tapped on a wooden bench 100 times until a consistent volume was reached. The initial and final volumes were represented as *V*_*o*_ and *V*_*f*_, respectively, and were used in the calculation of the compressibility (%) and Hausner's ratio [15]. The fixed height method was employed in the determination of the angle of repose. The computation was made using the respective equations as follows:

(1) $$\begin{array}{c} \text{angle of repose}={\text{Tan}}^{-1}\left(\frac{H}{0.5L}\right), \end{array}$$

(2) $$\begin{array}{c} \text{consolidated density}=\frac{mass\text{ of granules }}{\text{Vf}}, \end{array}$$

(3) $$\begin{array}{c} \text{fluff density}=\frac{\text{mass of granules }}{\text{Vo}}, \end{array}$$

(4) $$\begin{array}{c} \text{Hausner ratio}=\frac{\text{consolidated density}}{\text{fluff density}}, \end{array}$$

(5) $$\begin{array}{c} \left(\%\text{ compressibility}\right)=\frac{\text{consolidated density}-\text{fluff density}}{\text{consolidated density}}\times 100. \end{array}$$

#### 2.2.3. Investigation of the Drug-Excipient Compatibility

Bruker Alpha II Fourier transform infrared spectroscopy was employed in the scanning of the pectin, pectin-drug combination, and the drug samples individually across a wavenumber range of 4000 to 400 cm^−1^. The spectra were generated using OriginPro software 2024 (OriginLab Corporation, Northampton, Massachusetts, USA).

### 2.3. Evaluation of Tablet Properties

#### 2.3.1. Physicomechanical Properties

The evaluation of physicomechanical parameters, encompassing aspects such as weight uniformity, hardness, dimensions, disintegration, tensile strength, friability, crushing strength to friability ratio (CSFR), and the ratio of crushing strength to friability over disintegration time, was conducted in accordance with the methodologies reported by [16] and BP [17].

### 2.4. Calibration Curve for Paracetamol

A volume of 200 mL paracetamol solution of concentration 0.15% weight/volume (w/v) was prepared as a stock solution. Serial dilutions were performed to obtain solutions with concentrations ranging from 0.0004%, 0.0005%, 0.0006%, 0.0007%, and 0.0008% weight/volume. Spectrophotometric measurements were carried out to determine the absorbances, at the maximum wavelength of 245 nm. The calibration curve was generated by plotting the correlation between the concentration and the absorbance [17–19].

### 2.5. Assay

An equivalent weight of a dose in a single tablet was measured from a selection of ten crushed tablets. This was dissolved in 75 mL of phosphate buffer (pH of 5.8), and further diluted to 100 mL using the same buffer, and subsequently filtered. Serial dilutions were prepared, and the absorbance and drug content were computed based on the previously determined calibration curve and maximum wavelength, as per the methodology outlined by Owusu et al. [20] and Nyol and Gupta [21]. This process was replicated for each batch.

### 2.6. Dissolution Test

The in vitro dissolution analysis was conducted using the Veego UDA-8D machine and dissolution apparatus II, operating at a rotational speed of 50 rpm. From each batch, six tablets were randomly selected and individually placed in vessels containing 900 mL of phosphate buffer (pH 5.8) maintained at a temperature of 37 ± 0.5°C. At specified time intervals (5, 15, 30, 34, and 60 minutes), a 10 mL sample of media was extracted and subsequently filtered. From each filtrate, a volume of 0.50 mL was pipetted and diluted with phosphate buffer to a total volume of 50 mL. Spectrophotometric analysis of the resulting solution was performed at a wavelength of 245 nm. Phosphate buffer at a pH of 5.8 was used in the reference cell. The quantity of paracetamol was determined using the calibration curve. This procedure was replicated for all batches. The dissolution profile of paracetamol from each batch was established by plotting a graph depicting the percentage cumulative drug release as a function of time [17, 22, 23].

### 2.7. Data Analysis

The difference (*f*1) and similarity (*f*2) factors between the dissolution profiles were assessed by the model-independent comparison procedure reported by Adeleye et al. [24]. A (*f*2) value of 100 indicates that the test and reference profiles are completely similar, while a lesser number indicates an increasing dissimilarity between the two profiles [24]. The difference factor (*f*1) calculates the percentage disparity at each time point between the two curves, assessing their relative discrepancy. Similarity is established when (*f*1) is within 0 to 15 [25, 26]. GraphPad Prism version 8 (GraphPad Software, San Diego, California, USA) for Windows PC was utilized for the analytical process, and the mean ± standard deviation was computed for each dataset. The analysis involved the use of variance followed by Tukey's multiple comparison tests. A *p* value set at ≤0.05 was established as the threshold for statistical significance.

## 3. Results and Discussion

### 3.1. Precompression Analysis

The parameters utilized for assessing granular properties, encompassing fluff and consolidated densities, angle of repose, compressibility index and Hausner ratio, are presented in Table 2. The angle of repose serves as an indirect technique for gauging the flow characteristics of powders. As a general rule, values exceeding 50° are indicative of suboptimal flow, while those approaching a minimum of 25° are deemed to exhibit excellent flow [8, 27, 28]. All the angles of repose were lower than 50 and closer to 25 Hausner ratio between 0.96 ± 0.21–1.22 ± 0.02 and compressibility (%) of 7.69 ± 0.002–20.51 ± 0.002. This is suggestive that the physical blend of the powders with the pectin samples conferred good flow properties on the powder mix. Furthermore, the flow characteristics exhibited by the granules could be attributed to several factors, including the relatively smooth surface texture of the granules, minimal moisture content with the granules, an adequate distribution of particle sizes, and optimal particle shapes as reported by Kipo et al. [29] and Aulton and Taylor [8] for granules within optimal range of the parameters discussed.

### 3.2. Drug-Excipient Compatibility Testing Using FTIR

**Figure 1:**
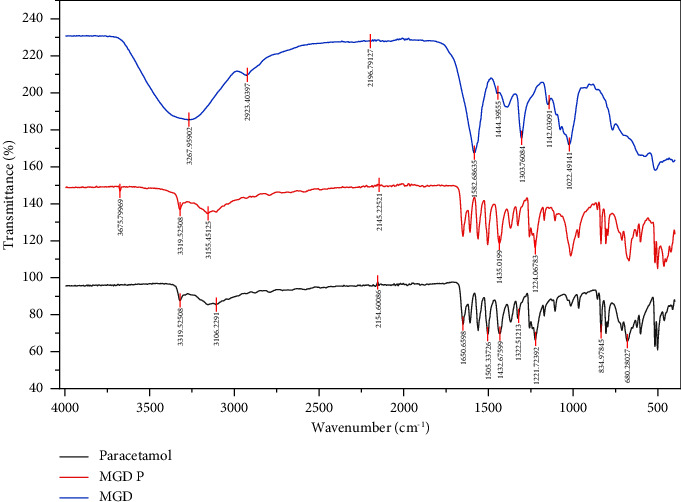
FTIR spectra of paracetamol (active), PPP (MGD), and the physical combination of paracetamol and PPP (MGD P).

**Figure 2:**
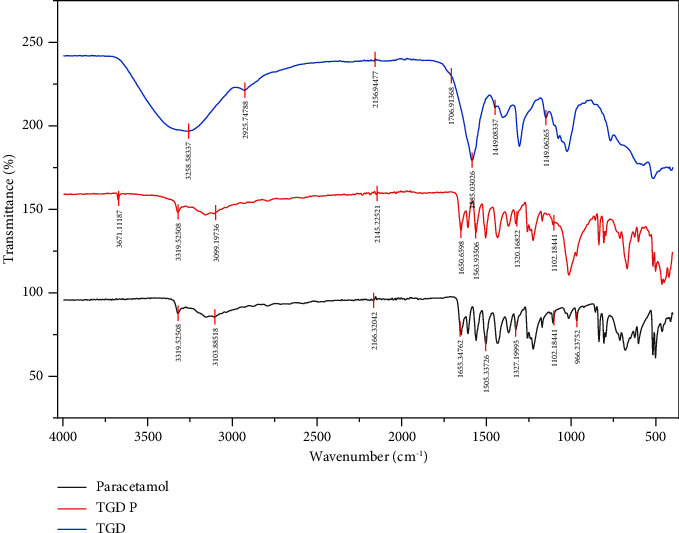
FTIR spectra of paracetamol (active), PPP (TGD), and the physical combination of paracetamol and PPP (TGD P).

The compatibility of pure paracetamol with the pectin extracts was investigated using FTIR spectroscopy. Representative spectra of pure paracetamol and its physical mixtures with pectin samples are presented in Figures 1 and 2. The structural analysis of pure paracetamol revealed the presence of functional groups (3322.03, 3159.39, 1561.11, and 1504.98 cm^−1^), which are characteristic features of the API used [30]. The results revealed no shifting of peaks, implying the stability and compatibility of the pectin and drug in the physical mixtures. The compatibility study between the drug and the excipient confirmed the stability of the physical mixture of paracetamol and various pectin extracts in the tablet form, suggesting no interaction between the drug and pectin samples. The spectra of all other physical mixtures of paracetamol-pectin are shown in the supplementary material section. Drug-excipient compatibility of the various varieties is shown in Supplementary FTIR.

### 3.3. Physical Parameters of Compressed Tablets

All the tablets that were formulated had weights varying from 645.40 ± 5.82 mg to 655.20 ± 8.31 mg (Table 3). In order for tablets to meet the uniformity of weight test criteria, no more than two individual tablets should deviate from the average weight by, more than ±5%, and none should deviate by more than double that percentage [17]. All the tablets formulated at various concentrations satisfied the uniformity of weight criteria, as indicated in Table 3. This can be ascribed to the consistent feeding of the powder into the die cavity, the favorable flow properties demonstrated by the powder mixture, the steady movement of the lower punch, and the consistency of the compressional force [15].

The dimensions of tablets, specifically its thickness and diameter, can exhibit variation without any alteration in the tablet's weight, attributable to the density of the granulation and the compression force applied. The thickness and diameter of all the formulations were consistent and comparable, with an average thickness and diameter ranging from 4.57 ± 0.17 to 4.85 ± 0.04 and 10.73 ± 0.38 to 12.03 ± 0.02, respectively (Table 3). These outcomes can be attributed to the uniform compressional force employed and the resemblances in the fluff and consolidated densities of the granules, which resulted in favorable flow properties of the granules [31].

**Figure 3:**
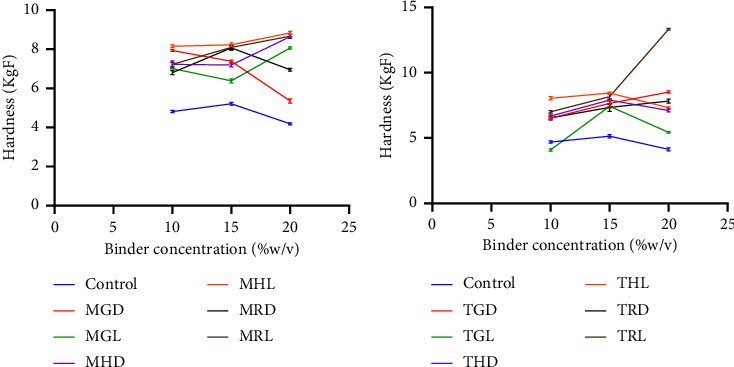
Effect of binder concentration on tablet hardness (a) Apem (M) (b) Apantu (T) at concentrations of 10% w/v, 15% w/v, and 20% w/v.

**Figure 4:**
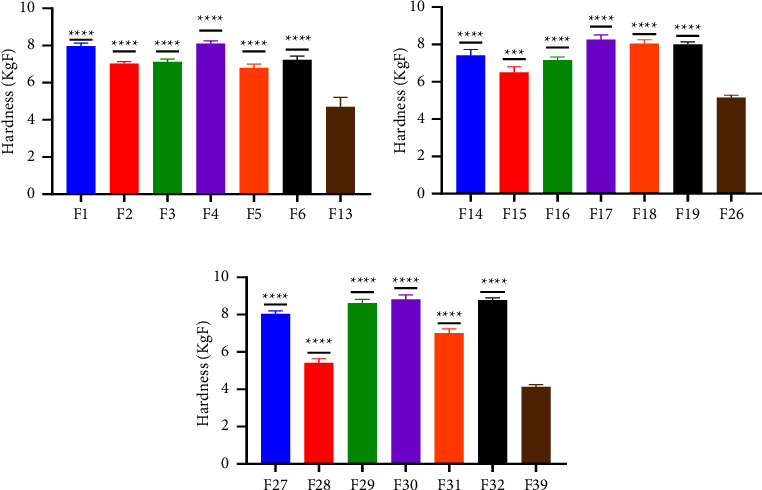
Comparative analysis of the crushing strength of pectin from the Apem variety as a binder at concentration (a)10% w/v, (b) 15% w/v, and (c) 20% w/v, respectively, and a standard binder using the Student's *t*-test. The values presented are mean ± SD (*n* = 6). Significance levels are denoted as follows:  ^*∗*^*p* ≤ 0.05,  ^*∗∗*^*p* ≤ 0.01,  ^*∗∗∗*^*p* ≤ 0.001,  ^*∗∗∗∗*^*p* ≤ 0.0001, and *p* > 0.05 not significant (ns).

**Figure 5:**
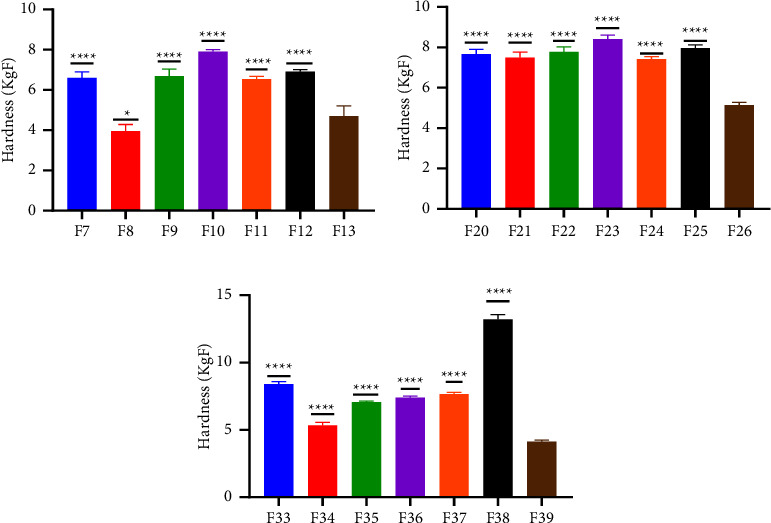
Comparative analysis of the crushing strength of pectin from the Apantu variety as binder at concentration (a) 10% w/w, (b) 15% w/w, and (c) 20% and a standard binder using Student's *t*-test. The values presented are mean ± SD (*n* = 6). Significance levels are denoted as follows:  ^*∗*^*p* ≤ 0.05,  ^*∗∗*^*p* ≤ 0.01,  ^*∗∗∗*^*p* ≤ 0.001,  ^*∗∗∗∗*^*p* ≤ 0.0001, and *p* > 0.05 not significant (ns).

Increasing the binder concentration in a tablet formulation can have several effects. It can increase the mechanical strength of the tablet; however, that is not a general rule as various polymers have a threshold for which they can function as intended [32]. In terms of friability, ELsabbagh et al. [33] found that tablets prepared with higher concentrations of binder showed the least friability percentage. However, these tablets also had the highest hardness value and the longest disintegration time. As per the British pharmacopoeia, 1% loss of tablet during transportation is permissible, and it was checked and the result showed that all batches for both the disintegrant and binder study had a friability within the range of 0.10 to 0.90% [17]. The friability values across the batches exhibited minimal, if any, deviation from their respective controls (Table 3). This implies the existence of rigorous control over tablet weight, an effective granulation process, a low moisture content, and a suitable binder concentration. The noted low percentages of friability suggest that the manufactured paracetamol tablets are capable of withstanding mechanical stress and impact during various phases such as processing, transportation, and handling [34]. In the context of hardness, a conventional is considered to require a minimum diametric crushing force of 3 kg/F [35]. All the tablets tested showed crushing forces greater than 3 kg/F, suggesting capacity to withstand wear and tear during transit and handling. Furthermore, the mechanical strength of tablet influences both the time it takes to disintegrate and the speed at which it dissolves. As the binder's concentration rises, so does the tablet's mechanical strength [32]. This characteristic was displayed by pectin from both varieties, affirming their appropriateness for binder use. However, the degree to which each type of pectin enhances tablet hardness when used as binders at higher concentrations varied. This was observed for both varieties of plantain cultivars (Figure 3). However, there was no increase in the mechanical strength of *F*27, *F*31, *F*33, *F*35, and *F*36 formulated with MGD, MRD, TGL, THD, and THL pectin, respectively, when the concentration was increased to 20%. This was also observed for tragacanth at 20%. Moreover, all the concentrations were significantly different from their respective control (*p* < 0.001) (Figures 4 and 5). Tablets prepared with pectin were significantly harder than those prepared with tragacanth (*p* < 0.05).

According to the British Pharmacopoeia [17], it is required that an immediate release paracetamol dosage unit has a minimum of 95% and a maximum of 105% of pure paracetamol (Table 3). Paracetamol concentration in all batches produced exhibited a range of 100.1 ± 0.01% to 102.2 ± 0.95%, suggesting that the tablets possess the necessary potency to effectively fulfil their therapeutic purpose.

### 3.4. Tensile Strength, Crushing Strength Friability Ratio (CSFR), and Crushing Strength Friability Ratio/Disintegration Time (CSFR/DT)

The tensile strength of a tablet is a crucial parameter. The tablet must possess sufficient mechanical strength to endure the pressures associated with handling, film coating, and packaging, yet it must also be sufficiently fragile to ensure the release of its contents upon administration [22, 32]. The tensile strength of the formulated tablets from both varieties ranged between 4.46 ± 0.15 and 14.73 ± 0.75 for all formulated batches using both varieties of pectin. With regard to the use of the pectin as a binder, it was observed that the tensile strength generally decreased from 10% to 20% for both control and test samples. Odeku and Itiola [36] reported that increasing the binder concentration in tablet formulation can lead to an increase in the tensile strength of a tablet because the binder helps hold the tablet together, providing the necessary strength. However, the effect of the binder on the tensile strength of a tablet can also depend on other factors such as the type of binder used, relative density of the tablets, and other processing variables [37].

CSFR provides a better measure of the tablet's mechanical strength compared to the crushing strength as it can do away with the weakness of the tablet due to friability [22]. High CSFR indicates that the tablet has sufficient crushing strength and low friability, which are desirable properties for tablet formulation. Low CSFR indicates that the tablet is either too soft or too brittle, which can affect drug content and efficacy [24, 38].  Table 4 shows a general increase in the CSFR values with an increase in the binder concentration. This was similar to the observation made by Adeleye et al. [24].

**Figure 6:**
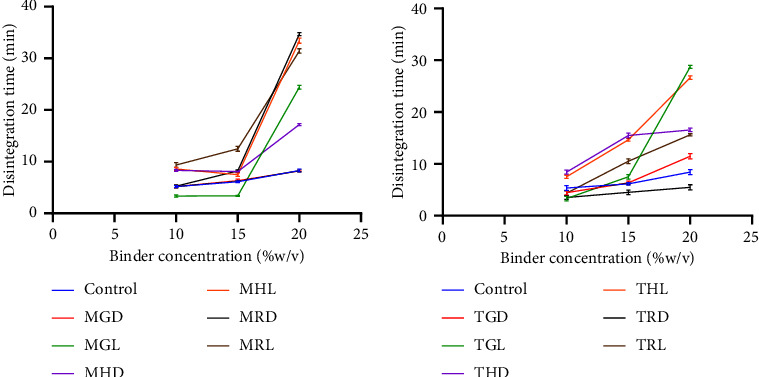
Effect of binder concentration on disintegration time (minutes) (a) Apem (M) (b) Apantu (T) at concentrations of 10% w/v, 15% w/v, and 20% w/v.

Table 4 shows the disintegration time of the various batches. The disintegration time ranged from 3.09 ± 0.07 minutes to 34.43 ± 0.02 minutes for all formulated batches. The disintegration time increased as the binder concentration increased (Figure 6). Tablet batches formulated with pectin at 10% and 15% as the binder all passed the disintegration test (*D*_*T*_ < 15 minutes). However, at 20% concentration, the tablets produced were compact and hard hence the failure to pass the disintegration test. Khan's [21] study demonstrated that an increase in both the concentration of the binder and the duration of granulation positively influenced the tablet's mechanical strength, while negatively impacting their rate of disintegration and dissolution. The same study indicated that granules made with the maximum binder concentration and duration of granulation led to tablets exhibiting the greatest resistance to crushing and the longest disintegration time. The tensile strength of the tablet could also decrease even though the concentration of the binder increases [39]. This is because higher concentrations of binder can lead to a more cohesive matrix within the tablet, which will make the matrix less flexible and more brittle, which can lead to a decrease in tensile strength. In a less cohesive matrix, stress can be distributed more evenly throughout the tablet, whereas in a cohesive matrix, stress may be concentrated in certain areas, making it more prone to break under applied pressure [40]. Conversely, the disintegration time of tablets typically increases with higher binder concentration. This is as a result of the resistance to penetration by fluid as the concentration of the binder increases [6].

The crushing strength-friability/disintegration time ratio is a key parameter in the evaluation of the balance between the binding and disintegration properties of a tablet as it is able to predict the balance between the hardness of the tablet and its ability to disintegrate. A high value of CSFR/DT indicates a better balance [41]. It was observed that the CSFR/DT for all formulations recorded peak CSFR/DT values at 10% w/v concentrations (Table 4). This indicates that a concentration of 10% w/v pectin as a binder might be the optimum to be used in the formulation of paracetamol conventional tablets. Additionally, no discernible pattern was noted for the CSFR/DT values as the binder concentration increased in both the control and test formulations. This observation aligns with the findings of Adetogun and Alebiowu [42]. The observed high DT and low CSFR/DT values might be attributed to the reduced lactose concentration which diminishes as the concentration of pectin increases. Lactose presence in the formulation facilitates water permeation into the tablet capillaries as it dissolves in the medium and therefore promotes faster disintegration by breaking the hydrogen bonds which exist between particles [43]. A summary of the raw dataset and postcompression analysis is shown in the Supplementary Ms. Excel file, “PPCD.”

### 3.5. In Vitro Drug Release

**Figure 7:**
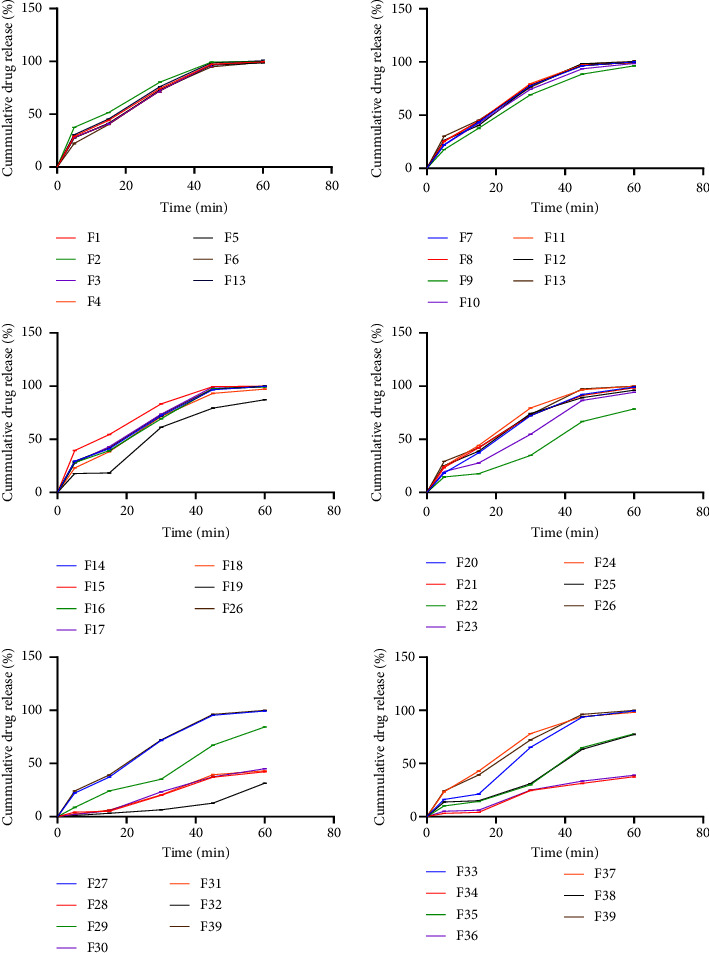
Dissolution profiles of tablets formulated with pectin from Apem (M) and Apantu (T) varieties as binder 10% w/w, 15% w/w, and 20% w/w, respectively, and a standard disintegrant (mean ± SD, *n* = 6).

The oral route of drug delivery is very effective and the most common in the treatment of various disease conditions. Dissolution testing assesses the impact of many pharmaceutical manufacturing factors, including mixing efficiency, granulation method, binding agent, and disintegrant effectiveness. It also helps forecast the behavior of the product inside the body [11]. The British Pharmacopoeia [17] stipulates that for immediate release tablets, a cumulative percentage of 75 or more (Diss, *Q* ≥ 75%) should be released within 45 minutes. Based on the results obtained, tablet batches *F*28, *F*29, *F*30, *F*31, *F*32, *F*34, *F*35, *F*36, and *F*38 which utilized a binder at a concentration of 20% w/w failed the dissolution test (Diss, *Q* < 75%) (Figure 7) [17]. These same batches failed the disintegration test (*D*_*T*_ > 15 minutes). It was observed that as the pectin concentration increased, particularly *F*28–*F*37, there was a notable change in the drug release. The tablets swelled which was an attribute of the pectin swelling behavior, leading to the formation of a gel layer. This gel layer or plug acted as a barrier to drug release, which is a common behavior observed in polymers at high concentrations [44]. This phenomenon was particularly evident in the 20% pectin concentration samples, where the gel layer's presence correlated with the higher tablet hardness and the consequent failure to meet the dissolution test criteria. It is important to consider tablet hardness when referring to drug release. An increase in the hardness of a tablet reduces the porosity, which may result in a decrease in water uptake and a slower rate of dissolution. Consequently, tablets should not be excessively soft or hard. Hard tablet could inhibit the dissolution necessary for accurate dosage, while an overly soft tablet could disintegrate too quickly and be prone to chipping or breaking during handling and transportation. Hence, employing these pectin extracts as binding agents in conventional tablets at concentrations surpassing 20% w/v will hinder the release of the thereby failure the criteria set by the British Pharmacopoeia. Nevertheless, they could be further exploited as prospective controlled-release agents at concentrations of 20% w/v and higher.

### 3.6. Model Independent Comparison of Dissolution Profiles (Difference *f*1 and Similarity *f*2 Factors)

The similarity and difference factors are shown in Table 5. The results show that the tablet batches formulated with pectin *F*19 and *F*23 as a binder at a concentration of 15% w/w were the only exceptions. Consequently, their release profiles are not similar to the reference binder used. This shows that plantain peel pectin extracted from different ripening stages, with their different concentrations, can be used as an alternative binder to tragacanth BP when developing immediate release tablets. Given the ease of access to these varieties of plantains in Ghana and the observed binding properties of their pectin suggest potential application in tablet formulation and further research could be undertaken to explore its use in developing bioequivalent products.

## 4. Conclusion

In conclusion, the prepared granules exhibited flow properties that were on par with the control formulations. The tablet batches formulated with varying concentrations of PPP met both compendial and noncompendial tests for uniformity of weight, content, tablet thickness and diameter, and friability. The mechanical strength of the tablet batches was assessed using CSFR and CSFR/DT ratio, and it was found that all batches generally had high values of CSFR and CSFR/DT ratio comparable to the control formulations. However, formulations (*F*28, *F*29, *F*30, *F*31, *F*32, *F*34, *F*35, *F*36, and *F*38) at a concentration of 20% w/w failed the disintegration test leading to a subsequent failure in the dissolution test. Interestingly, the pectin used in the formulation demonstrated potential controlled release characteristics at different concentration levels, suggesting its potential application in matrix tablets. This study opens up new avenues for the use of plantain peel pectin in pharmaceutical formulations.

## Figures and Tables

**Table 1:** Formula for the preparation of granules.

Formulation code	API (mg)	Disintegrant (mg)	Binder (%) w/v	Binder (%) w/v	Diluent (mg)	Lubricant (mg)
		Starch	Pectin	Tragacanth	Lactose	Talc
*F*1–*F*12	500	32.50	10	—	117.50	0.50
*F*13	500	32.50		10	117.50	0.50
*F*14–*F*25	500	48.75	15	—	101.25	0.50
*F*26	500	48.75		15	101.25	0.50
*F*27–*F*38	500	65.00	20		85.00	0.50
*F*39	500	65.00		20	85.00	0.50

**Table 2:** Flow properties of granules.

Formulation code	Fluff density	Consolidated density	Hausner's ratio	Compressibility (%)	Angle of repose
*F*1	0.42 ± 0.04	0.47 ± 0.02	1.11 ± 0.06	13.160 ± 0.001	18.73 ± 0.21
*F*2	0.43 ± 0.05	0.48 ± 0.01	1.11 ± 0.11	15.790 ± 0.001	19.03 ± 0.28
*F*3	0.40 ± 0.02	0.44 ± 0.03	1.09 ± 0.01	7.690 ± 0.002	17.88 ± 0.00
*F*4	0.44 ± 0.04	0.47 ± 0.00	1.06 ± 0.09	11.110 ± 0.001	17.10 ± 0.78
*F*5	0.42 ± 0.03	0.46 ± 0.03	1.11 ± 0.01	10.530 ± 0.003	15.95 ± 0.71
*F*6	0.46 ± 0.04	0.47 ± 0.02	1.03 ± 0.13	11.430 ± 0.001	16.39 ± 0.07
*F*7	0.44 ± 0.03	0.46 ± 0.01	1.06 ± 0.04	8.330 ± 0.002	17.35 ± 0.42
*F*8	0.56 ± 0.06	0.53 ± 0.09	0.97 ± 0.27	13.790 ± 0.002	16.16 ± 0.21
*F*9	0.41 ± 0.03	0.46 ± 0.04	1.12 ± 0.01	10.260 ± 0.003	19.98 ± 0.21
*F*10	0.41 ± 0.04	0.46 ± 0.03	1.12 ± 0.04	12.820 ± 0.000	18.73 ± 0.21
*F*11	0.48 ± 0.05	0.49 ± 0.04	1.03 ± 0.21	14.710 ± 0.003	19.03 ± 0.28
*F*12	0.49 ± 0.04	0.49 ± 0.03	1.02 ± 0.16	12.120 ± 0.001	17.88 ± 0.00
*F*13	0.41 ± 0.03	0.46 ± 0.04	1.12 ± 0.01	10.260 ± 0.003	16.39 ± 0.07
*F*14	0.41 ± 0.04	0.44 ± 0.00	1.07 ± 0.10	12.820 ± 0.000	19.65 ± 0.07
*F*15	0.48 ± 0.03	0.49 ± 0.01	1.03 ± 0.09	9.090 ± 0.001	20.32 ± 0.42
*F*16	0.47 ± 0.06	0.49 ± 0.04	1.04 ± 0.23	17.140 ± 0.002	17.88 ± 0.00
*F*17	0.44 ± 0.04	0.48 ± 0.02	1.11 ± 0.06	13.510 ± 0.002	20.32 ± 0.28
*F*18	0.41 ± 0.04	0.45 ± 0.01	1.09 ± 0.08	12.820 ± 0.000	18.43 ± 0.28
*F*19	0.43 ± 0.03	0.46 ± 0.01	1.08 ± 0.06	10.810 ± 0.001	16.62 ± 0.14
*F*20	0.43 ± 0.06	0.44 ± 0.04	1.05 ± 0.23	17.950 ± 0.001	15.95 ± 0.07
*F*21	0.44 ± 0.03	0.46 ± 0.01	1.06 ± 0.04	8.330 ± 0.002	16.86 ± 0.64
*F*22	0.47 ± 0.04	0.47 ± 0.04	1.01 ± 0.18	11.760 ± 0.003	19.03 ± 0.35
*F*23	0.45 ± 0.05	0.48 ± 0.00	1.08 ± 0.11	13.890 ± 0.001	19.65 ± 0.07
*F*24	0.46 ± 0.04	0.47 ± 0.02	1.03 ± 0.13	11.430 ± 0.001	20.32 ± 0.42
*F*25	0.45 ± 0.07	0.55 ± 0.07	1.22 ± 0.02	18.920 ± 0.001	17.88 ± 0.00
*F*26	0.41 ± 0.04	0.45 ± 0.01	1.09 ± 0.08	12.820 ± 0.000	18.43 ± 0.28
*F*27	0.47 ± 0.04	0.47 ± 0.04	1.01 ± 0.18	11.760 ± 0.003	16.62 ± 0.14
*F*28	0.43 ± 0.03	0.45 ± 0.01	1.05 ± 0.11	10.810 ± 0.001	19.33 ± 0.07
*F*29	0.43 ± 0.05	0.46 ± 0.01	1.08 ± 0.15	15.790 ± 0.000	18.43 ± 0.14
*F*30	0.44 ± 0.04	0.47 ± 0.00	1.08 ± 0.11	13.510 ± 0.002	17.88 ± 0.21
*F*31	0.46 ± 0.04	0.48 ± 0.01	1.05 ± 0.11	11.430 ± 0.001	21.80 ± 1.41
*F*32	0.43 ± 0.07	0.46 ± 0.03	1.08 ± 0.24	20.510 ± 0.002	17.35 ± 0.28
*F*33	0.42 ± 0.04	0.48 ± 0.03	1.13 ± 0.04	13.160 ± 0.001	17.10 ± 0.07
*F*34	0.44 ± 0.04	0.49 ± 0.03	1.11 ± 0.02	11.110 ± 0.001	15.73 ± 0.14
*F*35	0.43 ± 0.05	0.47 ± 0.00	1.09 ± 0.13	15.790 ± 0.000	14.93 ± 1.41
*F*36	0.45 ± 0.05	0.46 ± 0.03	1.04 ± 0.18	13.890 ± 0.001	20.67 ± 0.42
*F*37	0.48 ± 0.05	0.49 ± 0.04	1.03 ± 0.21	14.710 ± 0.003	19.33 ± 0.07
*F*38	0.55 ± 0.04	0.52 ± 0.08	0.96 ± 0.21	10.340 ± 0.003	18.43 ± 0.14
*F*39	0.46 ± 0.05	0.48 ± 0.03	1.04 ± 0.18	14.290 ± 0.003	17.88 ± 0.14

**Table 3:** Physical parameters of compressed tablets.

Formulation code	Average weight *n* = 20	Assay (%)	Hardness (Kg/F) *n* = 6	Friability (%)	Tablet thickness (mm) *n* = 6	Tablet diameter (mm) *n* = 6
*F*1	650.30 ± 5.13	100.20 ± 0.10	6.80 ± 0.20	0.70 ± 0.01	4.71 ± 0.03	11.96 ± 0.02
*F*2	649.30 ± 3.70	100.80 ± 0.62	7.23 ± 0.21	0.50 ± 0.01	4.61 ± 0.11	11.96 ± 0.03
*F*3	650.10 ± 5.12	101.80 ± 1.68	7.42 ± 0.12	0.40 ± 0.01	4.75 ± 0.15	11.99 ± 0.03
*F*4	648.80 ± 4.09	100.10 ± 0.01	7.97 ± 0.16	0.30 ± 0.01	4.49 ± 0.09	10.73 ± 0.38
*F*5	650.40 ± 4.91	100.60 ± 0.49	7.42 ± 0.31	0.80 ± 0.01	4.73 ± 0.07	12.00 ± 0.01
*F*6	645.50 ± 3.35	100.80 ± 0.95	5.42 ± 0.22	0.70 ± 0.00	4.69 ± 0.02	11.97 ± 0.05
*F*7	649.40 ± 7.14	101.20 ± 1.09	7.02 ± 0.12	0.40 ± 0.01	4.76 ± 0.01	11.95 ± 0.04
*F*8	646.40 ± 3.71	101.60 ± 1.07	6.50 ± 0.30	0.80 ± 0.01	4.74 ± 0.01	11.91 ± 0.05
*F*9	648.90 ± 2.81	100.30 ± 0.72	8.05 ± 0.15	0.30 ± 0.00	4.69 ± 0.08	11.93 ± 0.07
*F*10	650.40 ± 2.60	101.40 ± 0.19	7.12 ± 0.15	0.90 ± 0.01	4.63 ± 0.13	11.98 ± 0.02
*F*11	651.90 ± 3.75	101.30 ± 0.19	7.15 ± 0.18	0.80 ± 0.01	4.70 ± 0.09	11.99 ± 0.03
*F*12	653.20 ± 3.38	101.30 ± 0.19	8.61 ± 0.21	0.70 ± 0.00	4.63 ± 0.05	11.94 ± 0.05
*F*13	647.90 ± 4.63	100.10 ± 0.00	4.78 ± 0.00	0.50 ± 0.00	4.66 ± 0.00	11.90 ± 0.00
*F*14	652.10 ± 2.40	101.00 ± 1.09	8.26 ± 0.25	0.40 ± 0.01	4.57 ± 0.17	11.92 ± 0.06
*F*15	650.50 ± 6.35	101.10 ± 0.88	8.82 ± 0.24	0.30 ± 0.00	4.68 ± 0.09	11.96 ± 0.05
*F*16	650.30 ± 5.13	100.20 ± 0.10	6.80 ± 0.20	0.70 ± 0.01	4.71 ± 0.03	11.96 ± 0.02
*F*17	648.90 ± 3.86	100.50 ± 0.67	8.04 ± 0.21	0.40 ± 0.01	4.76 ± 0.01	11.99 ± 0.03
*F*18	652.10 ± 1.59	101.00 ± 1.07	7.00 ± 0.24	0.60 ± 0.00	4.69 ± 0.05	11.96 ± 0.04
*F*19	649.30 ± 3.70	100.80 ± 0.62	7.23 ± 0.21	0.50 ± 0.01	4.61 ± 0.11	11.96 ± 0.03
*F*20	650.60 ± 3.30	100.90 ± 0.91	8.01 ± 0.13	0.50 ± 0.01	4.49 ± 0.09	11.92 ± 0.06
*F*21	652.10 ± 6.32	101.50 ± 1.15	8.77 ± 0.12	0.30 ± 0.00	4.66 ± 0.11	11.95 ± 0.04
*F*22	654.40 ± 4.78	100.80 ± 0.00	6.60 ± 0.30	0.60 ± 0.01	4.80 ± 0.04	11.97 ± 0.07
*F*23	649.40 ± 2.43	101.20 ± 0.43	7.66 ± 0.24	0.40 ± 0.01	4.62 ± 0.02	12.01 ± 0.05
*F*24	651.40 ± 5.62	101.40 ± 1.22	8.40 ± 0.19	0.40 ± 0.00	4.85 ± 0.04	11.99 ± 0.01
*F*25	652.60 ± 6.98	101.40 ± 1.22	3.96 ± 0.32	0.20 ± 0.01	4.74 ± 0.13	11.93 ± 0.06
*F*26	645.40 ± 5.82	103.40 ± 0.00	5.17 ± 0.00	0.40 ± 0.00	4.69 ± 0.00	11.97 ± 0.00
*F*27	649.10 ± 4.08	100.70 ± 0.81	5.34 ± 0.22	0.30 ± 0.00	4.74 ± 0.05	12.03 ± 0.02
*F*28	648.50 ± 4.99	101.30 ± 0.56	6.70 ± 0.35	0.20 ± 0.01	4.76 ± 0.04	11.97 ± 0.07
*F*29	652.80 ± 5.59	100.90 ± 0.64	7.77 ± 0.25	0.20 ± 0.01	4.74 ± 0.04	11.99 ± 0.03
*F*30	652.00 ± 2.87	100.50 ± 0.52	7.07 ± 0.07	0.30 ± 0.00	4.68 ± 0.11	12.02 ± 0.06
*F*31	655.20 ± 8.31	100.50 ± 0.62	7.91 ± 0.10	0.30 ± 0.01	4.80 ± 0.07	12.01 ± 0.01
*F*32	650.70 ± 2.43	100.50 ± 0.26	8.41 ± 0.19	0.40 ± 0.01	4.75 ± 0.12	12.02 ± 0.05
*F*33	652.50 ± 5.16	101.10 ± 1.48	7.40 ± 0.10	0.40 ± 0.00	4.74 ± 0.09	11.98 ± 0.02
*F*34	654.60 ± 2.52	101.60 ± 1.07	6.54 ± 0.14	0.30 ± 0.01	4.71 ± 0.14	11.94 ± 0.07
*F*35	650.10 ± 5.12	101.80 ± 1.68	7.42 ± 0.12	0.40 ± 0.01	4.75 ± 0.15	11.99 ± 0.03
*F*36	653.30 ± 5.03	101.80 ± 1.68	7.65 ± 0.14	0.20 ± 0.00	4.70 ± 0.10	12.03 ± 0.01
*F*37	654.00 ± 4.57	102.20 ± 0.95	6.91 ± 0.10	0.10 ± 0.01	4.69 ± 0.12	11.99 ± 0.09
*F*38	648.80 ± 4.53	102.20 ± 0.95	7.97 ± 0.15	0.20 ± 0.01	4.75 ± 0.04	11.98 ± 0.02
*F*39	647.70 ± 3.18	102.20 ± 0.00	4.16 ± 0.00	0.50 ± 0.00	4.70 ± 0.00	11.99 ± 0.00

**Table 4:** Tensile strength, crushing strength-friability ratio (CSFR), and crushing strength-friability/disintegration time (CSFR/DT) of paracetamol tablets.

Formulation code	Tensile strength (kN/cm^2^)	CSFR	CSFR/DT	Disintegration time (DT) (minutes)
*F*1	9.24 ± 0.32	27.02	4.93	5.48 ± 0.08
*F*2	8.60 ± 1.16	17.77	4.79	3.71 ± 0.30
*F*3	14.73 ± 0.75	7.95	0.98	8.15 ± 0.05
*F*4	10.53 ± 0.20	13.62	1.64	8.33 ± 0.16
*F*5	8.33 ± 1.09	9.78	1.84	5.33 ± 0.09
*F*6	6.15 ± 0.82	14.60	1.58	9.22 ± 0.05
*F*7	7.85 ± 0.21	11.09	2.35	4.72 ± 0.25
*F*8	7.33 ± 0.89	20.32	6.58	3.09 ± 0.07
*F*9	9.16 ± 0.56	34.34	4.10	8.38 ± 0.18
*F*10	8.17 ± 0.30	26.82	3.61	7.44 ± 0.02
*F*11	8.08 ± 0.10	22.16	6.98	3.17 ± 0.03
*F*12	9.91 ± 0.64	72.74	17.41	4.18 ± 0.04
*F*13	5.49 ± 0.00	9.56	1.87	5.11 ± 0.00
*F*14	9.66 ± 0.68	9.33	1.48	6.33 ± 0.12
*F*15	10.04 ± 0.53	8.18	2.09	3.92 ± 0.94
*F*16	7.68 ± 0.35	8.99	1.10	8.15 ± 0.04
*F*17	8.97 ± 1.07	20.92	2.88	7.27 ± 0.07
*F*18	7.95 ± 0.63	20.36	2.42	8.42 ± 0.10
*F*19	8.34 ± 0.16	16.18	1.34	12.1 ± 0.09
*F*20	9.52 ± 0.63	19.40	3.12	6.23 ± 0.19
*F*21	10.02 ± 0.27	18.97	2.57	7.37 ± 0.05
*F*22	7.31 ± 0.12	39.86	2.57	15.50 ± 0.08
*F*23	8.79 ± 0.91	21.30	1.47	14.46 ± 0.05
*F*24	9.20 ± 1.49	18.78	4.69	4.00 ± 0.01
*F*25	4.46 ± 0.15	40.86	4.05	10.10 ± 0.06
*F*26	5.86 ± 0.00	12.93	2.14	6.04 ± 0.00
*F*27	5.96 ± 1.07	7.80	0.95	8.19 ± 0.10
*F*28	7.48 ± 0.15	27.29	1.11	24.69 ± 0.27
*F*29	8.70 ± 0.00	11.56	0.67	17.21 ± 0.08
*F*30	8.01 ± 1.68	29.91	0.90	33.21 ± 0.07
*F*31	8.74 ± 0.10	11.76	0.34	34.43 ± 0.02
*F*32	9.39 ± 0.44	29.74	0.95	31.34 ± 0.16
*F*33	8.30 ± 1.68	21.27	1.87	11.40 ± 0.08
*F*34	7.40 ± 0.14	18.10	0.64	28.36 ± 0.15
*F*35	8.29 ± 1.23	23.98	1.48	16.22 ± 0.04
*F*36	8.62 ± 0.96	18.73	0.71	26.40 ± 0.36
*F*37	7.82 ± 0.10	39.25	7.24	5.42 ± 0.14
*F*38	8.91 ± 0.33	67.74	4.38	15.47 ± 0.07
*F*39	4.70 ± 0.00	8.32	1.00	8.34 ± 0.00

**Table 5:** Difference (*f*1) and similarity (*f*2) factors of tablet batches formulated with pectin sample as the binder.

Formulation code	Difference factor (*f*1)	Similarity factor (*f*2)	Comment
*F*1	1.79	90.26	Similar
*F*2	5.40	68.52	Similar
*F*3	4.64	73.21	Similar
*F*4	3.30	78.78	Similar
*F*5	4.51	75.20	Similar
*F*6	6.27	67.25	Similar
*F*7	4.26	71.49	Similar
*F*8	3.24	79.20	Similar
*F*9	12.00	54.38	Similar
*F*10	6.11	66.72	Similar
*F*11	3.72	76.44	Similar
*F*12	3.70	75.65	Similar
*F*14	0.97	95.52	Similar
*F*15	9.95	55.73	Similar
*F*16	2.63	81.40	Similar
*F*17	1.32	93.04	Similar
*F*18	5.73	70.28	Similar
*F*19	23.01	41.07	Dissimilar
*F*20	6.53	63.55	Similar
*F*21	4.25	71.81	Similar
*F*23	17.23	46.92	Dissimilar
*F*24	3.95	73.44	Similar
*F*25	5.93	67.80	Similar
*F*27	1.82	89.64	Similar
*F*33	11.06	53.07	Similar
*F*37	4.50	74.22	Similar

## Data Availability

The supporting data for the results of the study are included in the article and are also available from the corresponding author.

## Supplementary Materials

Supplementary Materials Supplementary 1. PPCD: Pre- and postcompression analysis of the formulated tablets. Supplementary 2. FTIR Supplementary: Drug-excipient compatibility IR of the PPP varieties and paracetamol.
