# Supplementary material for: Biogenic Waste from Two Varieties of Plantain in Ghana Contain Pectin with Potential Binding Properties in Conventional Tablets
Source: ScientificWorldJournal. 2024 Jun 17;2024:5461358. doi: 10.1155/2024/5461358 (PMC11196187; doi:10.1155/2024/5461358)
Supplement: Supplementary Materials — Supplementary 1. PPCD: Pre- and postcompression analysis of the formulated tablets. Supplementary 2. FTIR Supplementary: Drug-excipient compatibility IR of the PPP varieties and paracetamol. [file 5461358.f1.zip › FTIR Supplementary (1).docx]

Supplementary materials

Figure 1. FTIR spectra of paracetamol (active), PPP (MGL), and the physical combination of paracetamol and PPP (MGL P)

Figure 2. FTIR spectra of paracetamol (active), PPP (MHD), and the physical combination of paracetamol and PPP (MHD P)

Figure 3. FTIR spectra of paracetamol (active), PPP (MHL), and the physical combination of paracetamol and PPP (MHL P)

Figure 4. FTIR spectra of paracetamol (active), PPP (MRD), and the physical combination of paracetamol and PPP (MRD P)

Figure 5. FTIR spectra of paracetamol (active), PPP (MRL), and the physical combination of paracetamol and PPP (MRL P)

Figure 6. FTIR spectra of paracetamol (active), PPP (TGL), and the physical combination of paracetamol and PPP (TGL P)

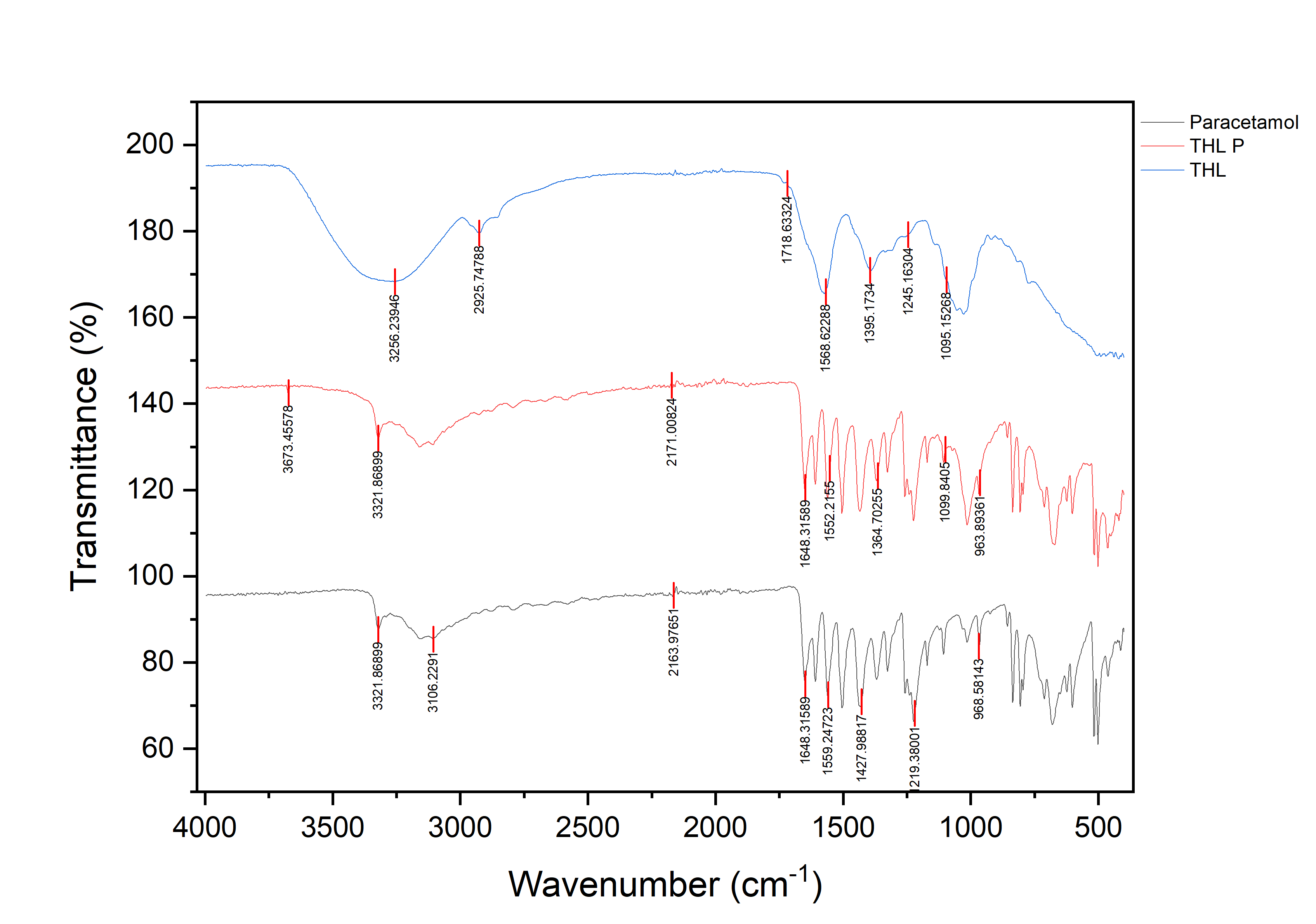
Figure 7. FTIR spectra of paracetamol (active), PPP (THD), and the physical combination of paracetamol and PPP (THD P)

Figure 8. FTIR spectra of paracetamol (active), PPP (THL), and the physical combination of paracetamol and PPP (THL P)

Figure 9. FTIR spectra of paracetamol (active), PPP (TRD), and the physical combination of paracetamol and PPP (TRD P)
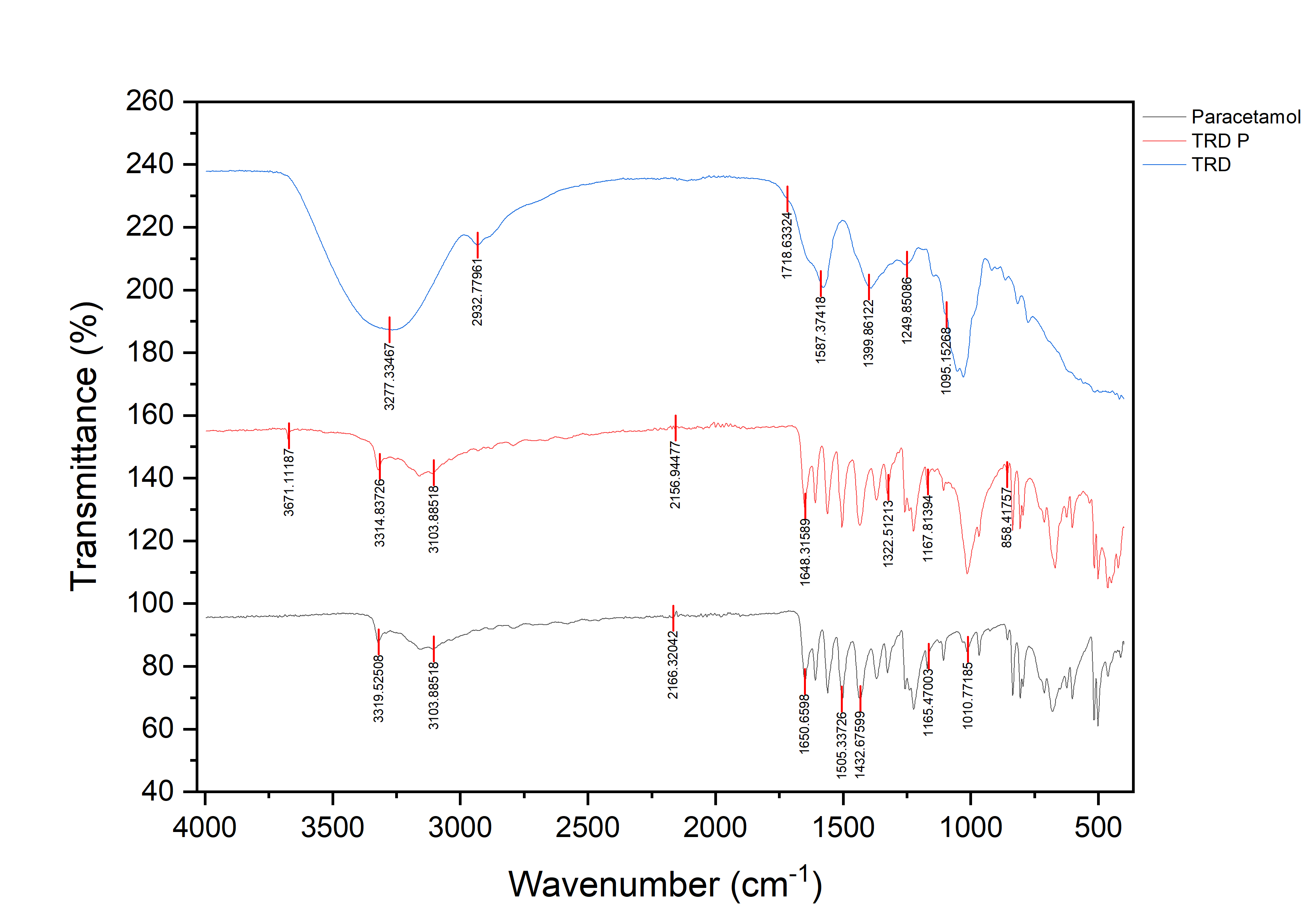


Figure 10. FTIR spectra of paracetamol (active), PPP (TRL), and the physical combination of paracetamol and PPP (TRL P)
