# Supplementary material for: Biogenic Waste from Two Varieties of Plantain in Ghana Contain Pectin with Potential Binding Properties in Conventional Tablets
Source: ScientificWorldJournal. 2024 Jun 17;2024:5461358. doi: 10.1155/2024/5461358 (PMC11196187; doi:10.1155/2024/5461358)
Supplement: Supplementary Materials — Supplementary 1. PPCD: Pre- and postcompression analysis of the formulated tablets. Supplementary 2. FTIR Supplementary: Drug-excipient compatibility IR of the PPP varieties and paracetamol. [file 5461358.f1.zip › Supplementary Description.docx]

Supplementary Material:

PPCD: Pre and post compression analysis of the formulated tablets

NOTE-PLEASE BE INFORMED THAT THE SUPPLEMENTARY FILE [PPCD] ARE UPLOADED UNDER FIGURES & TABLES SECTION.
