# Supplementary figures and images for: Biogenic Waste from Two Varieties of Plantain in Ghana Contain Pectin with Potential Binding Properties in Conventional Tablets
Source: ScientificWorldJournal. 2024 Jun 17;2024:5461358. doi: 10.1155/2024/5461358 (PMC11196187; doi:10.1155/2024/5461358)

MRL 10%

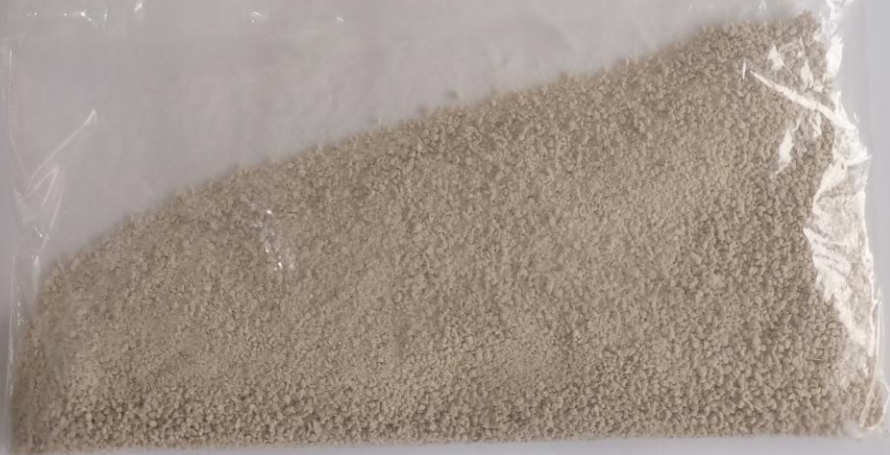

MGD 15%

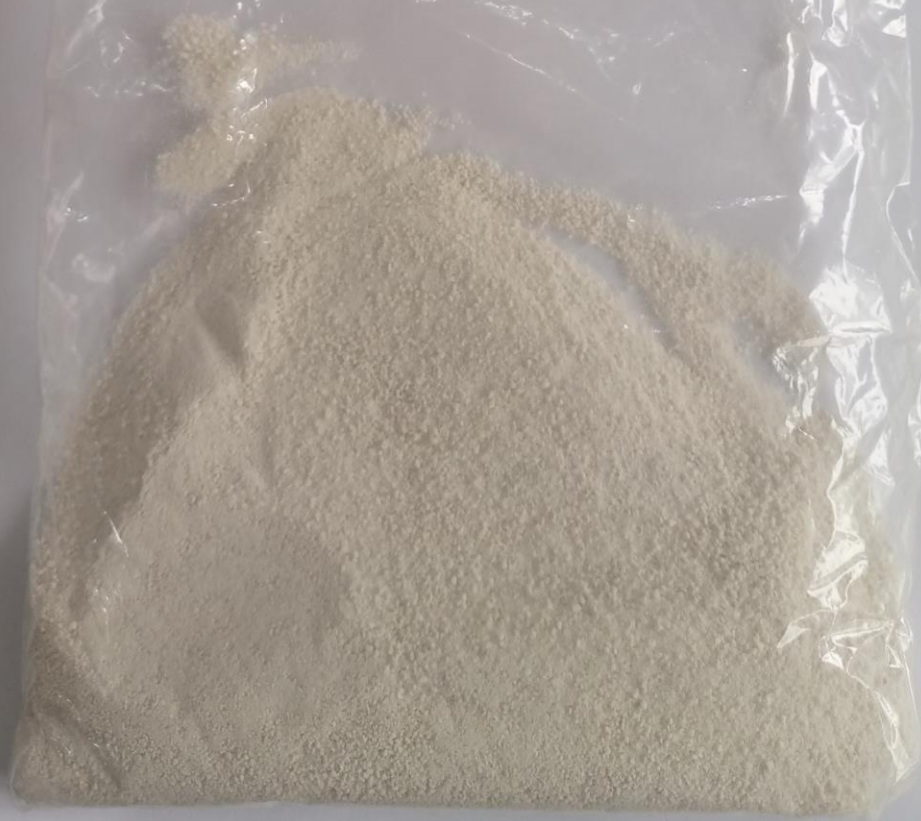

MGD 209

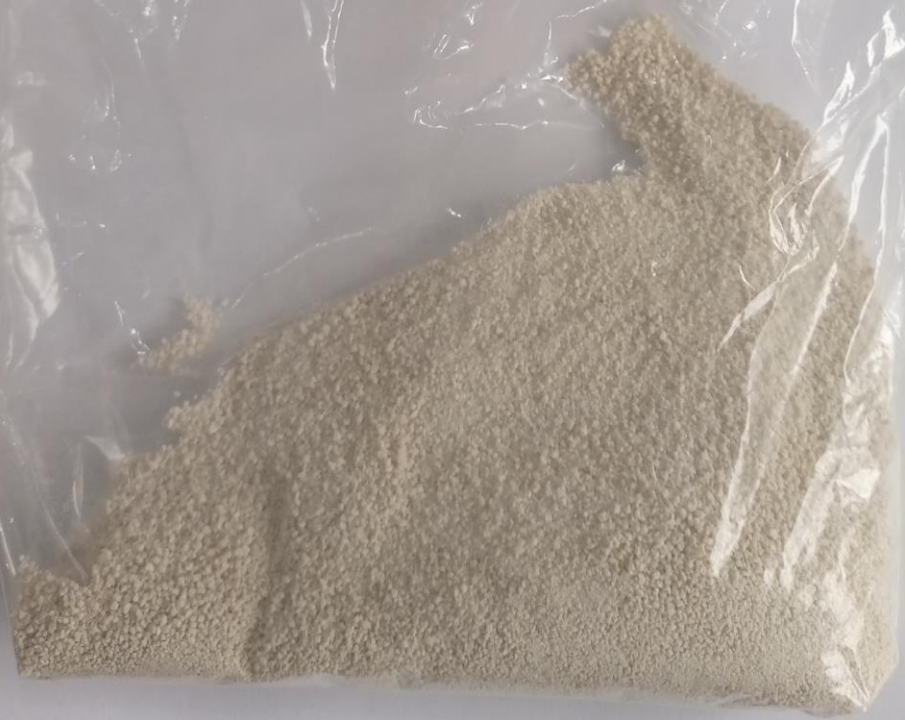

MRD 206

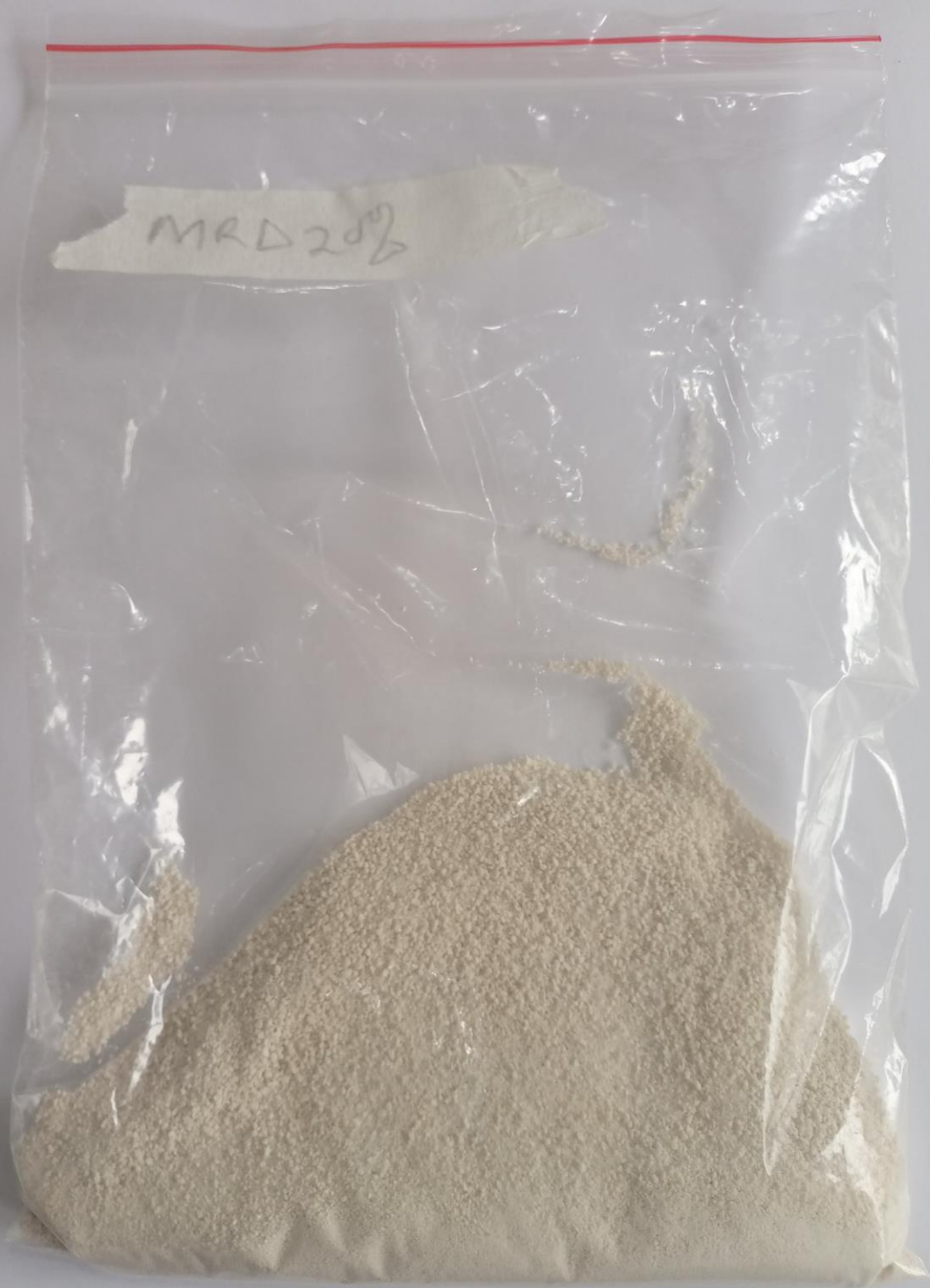

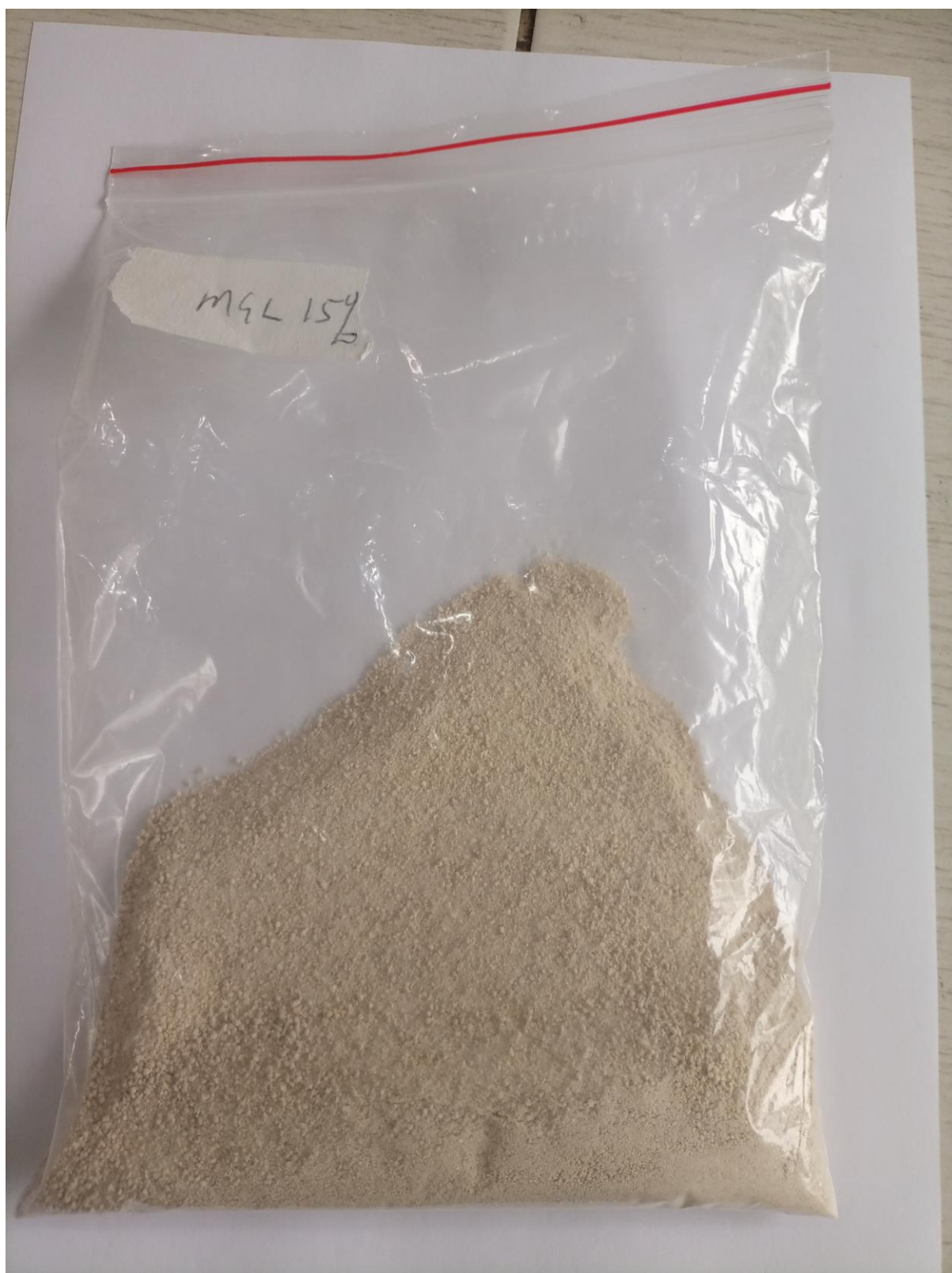

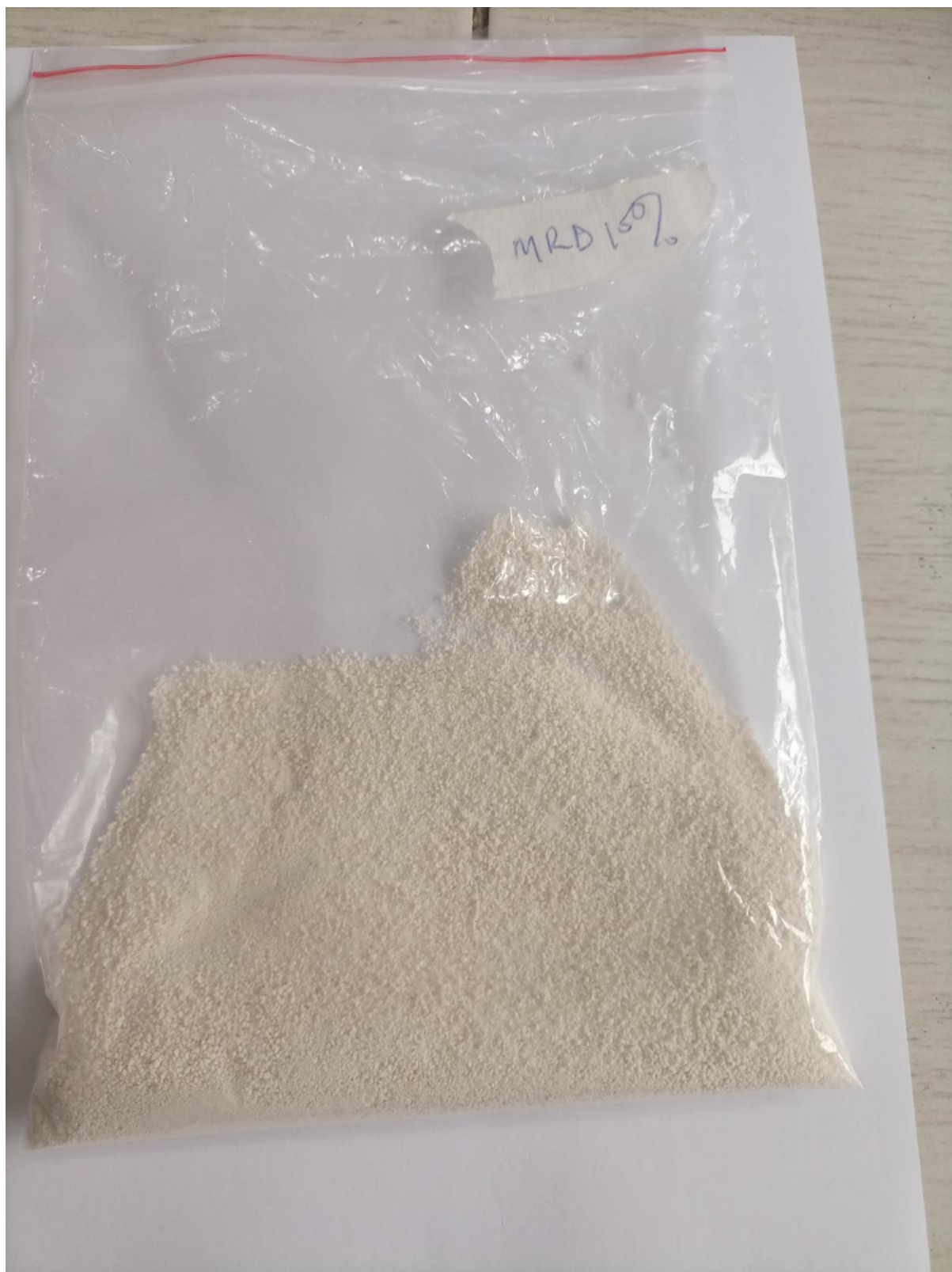

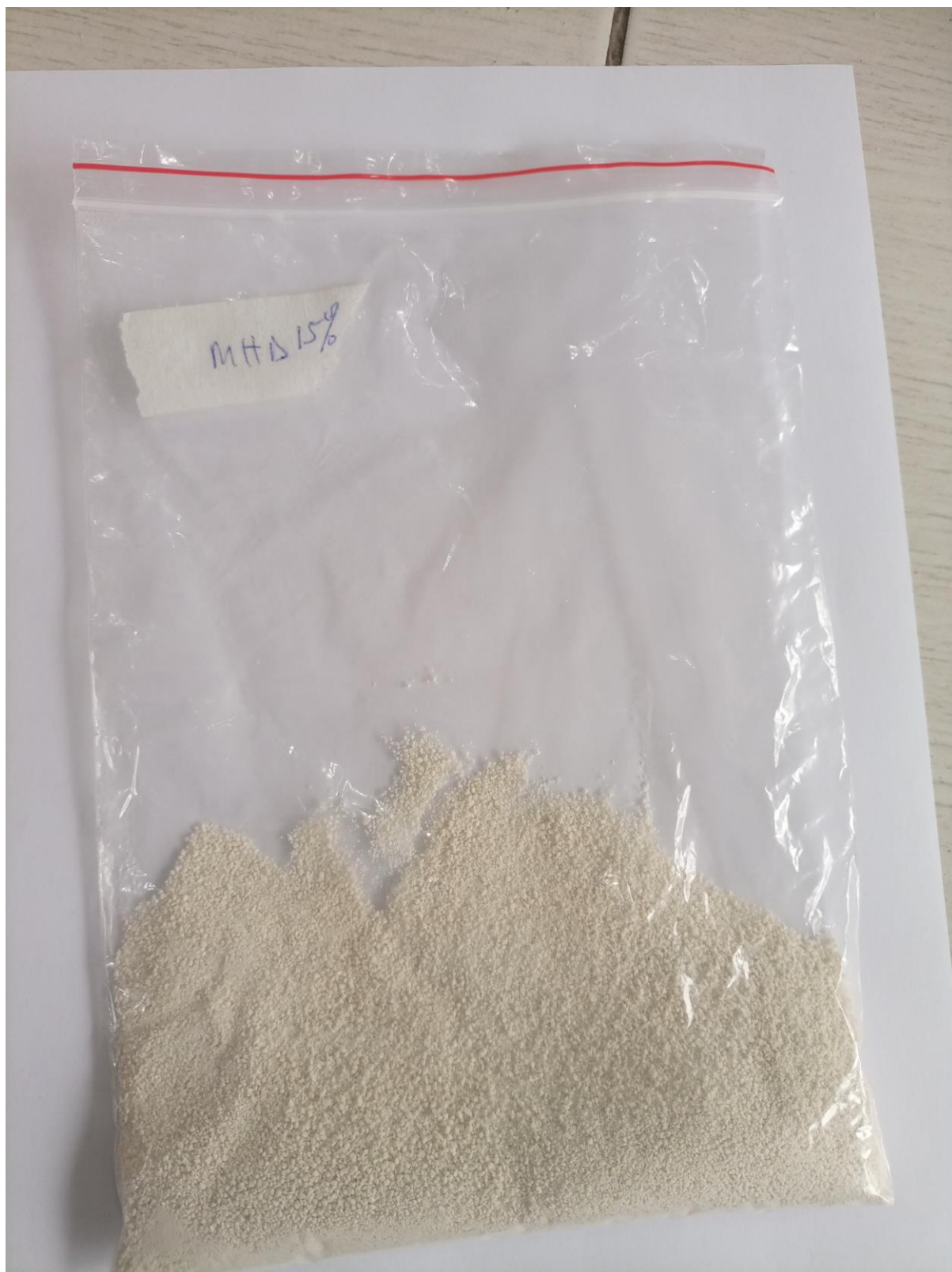

MGL 187

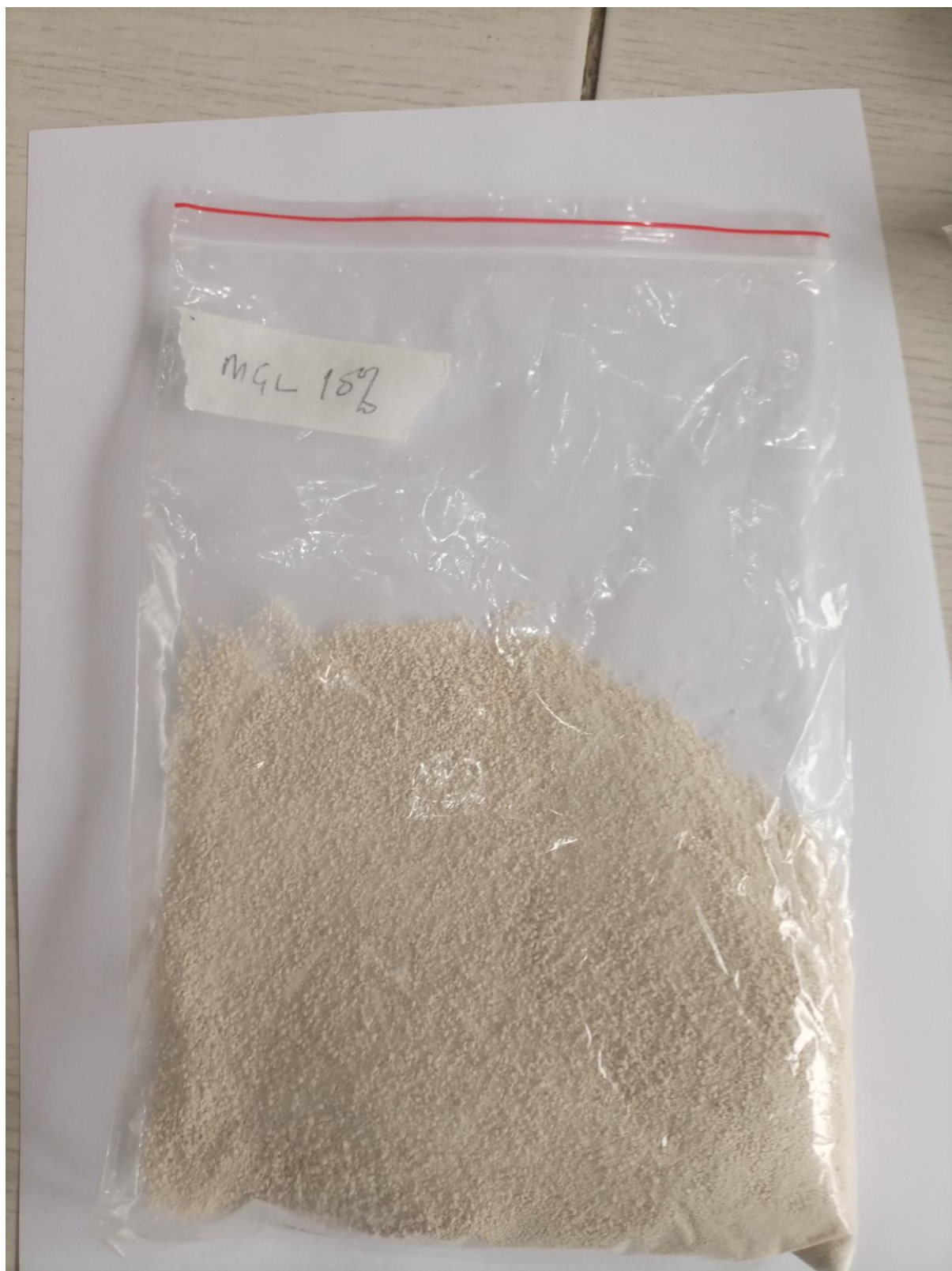

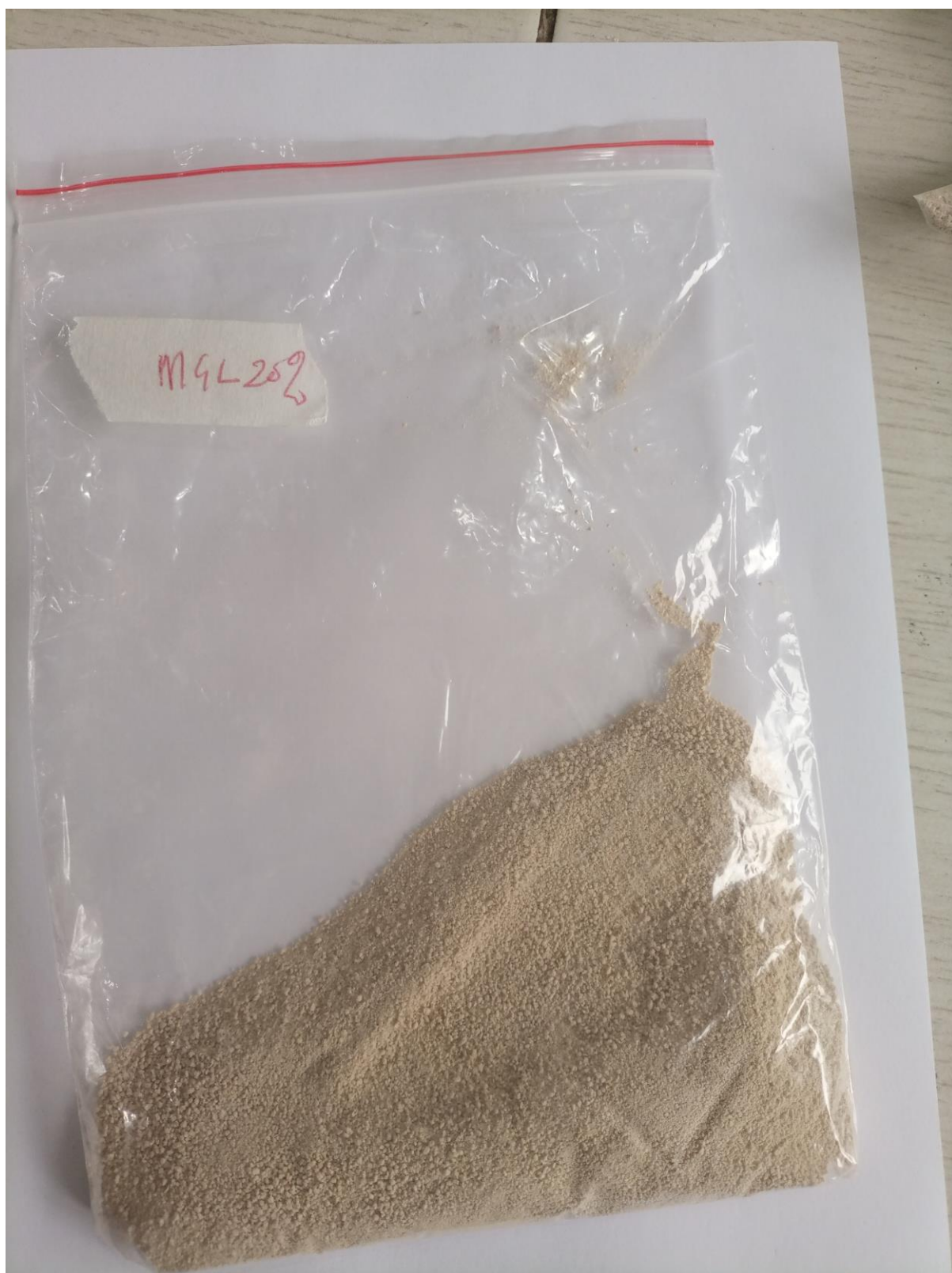

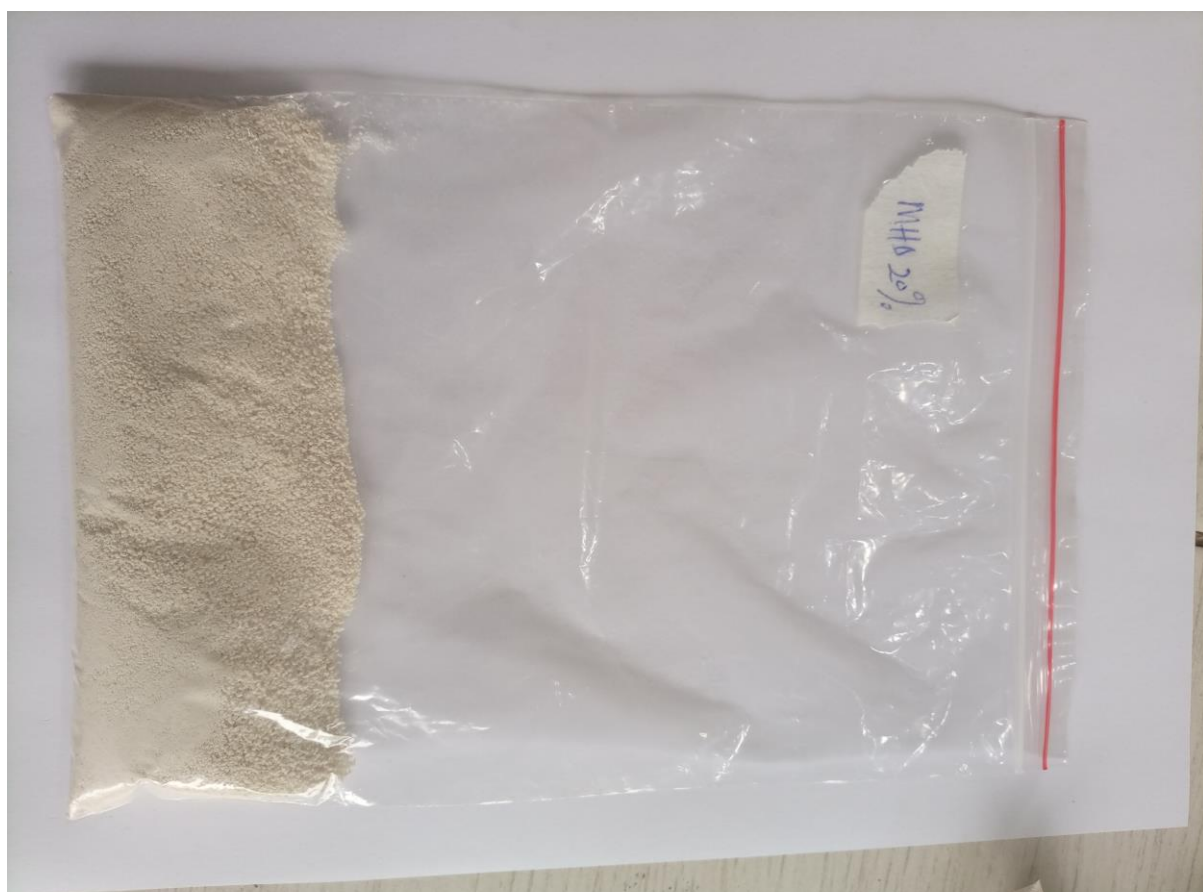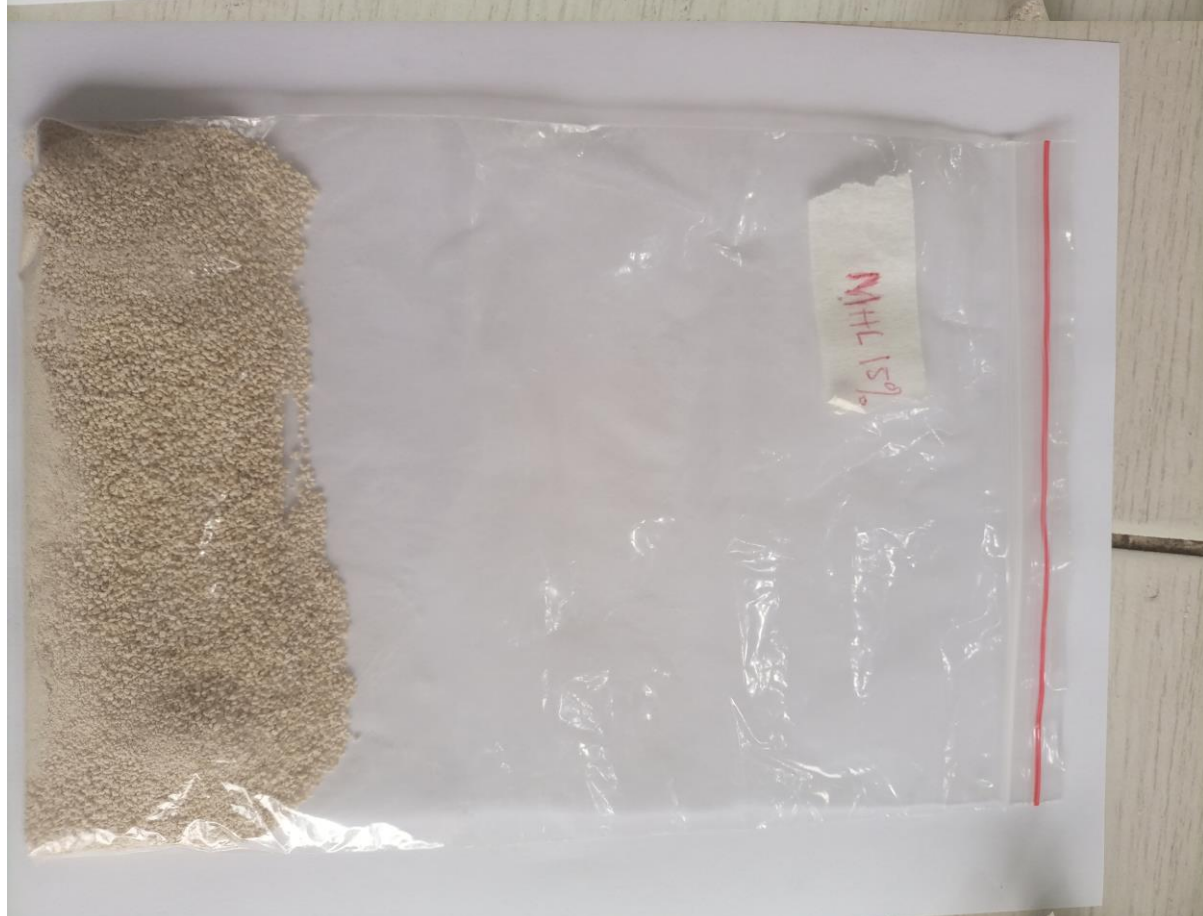

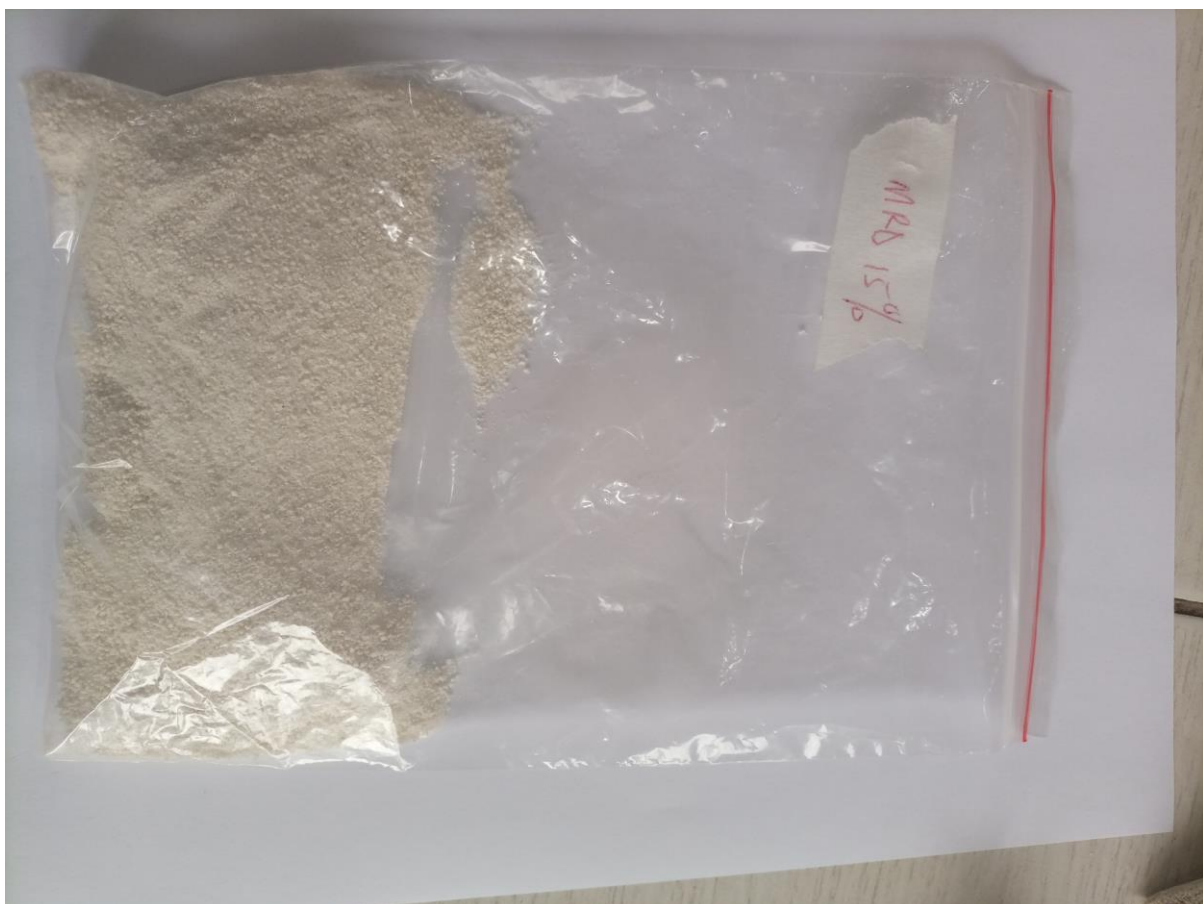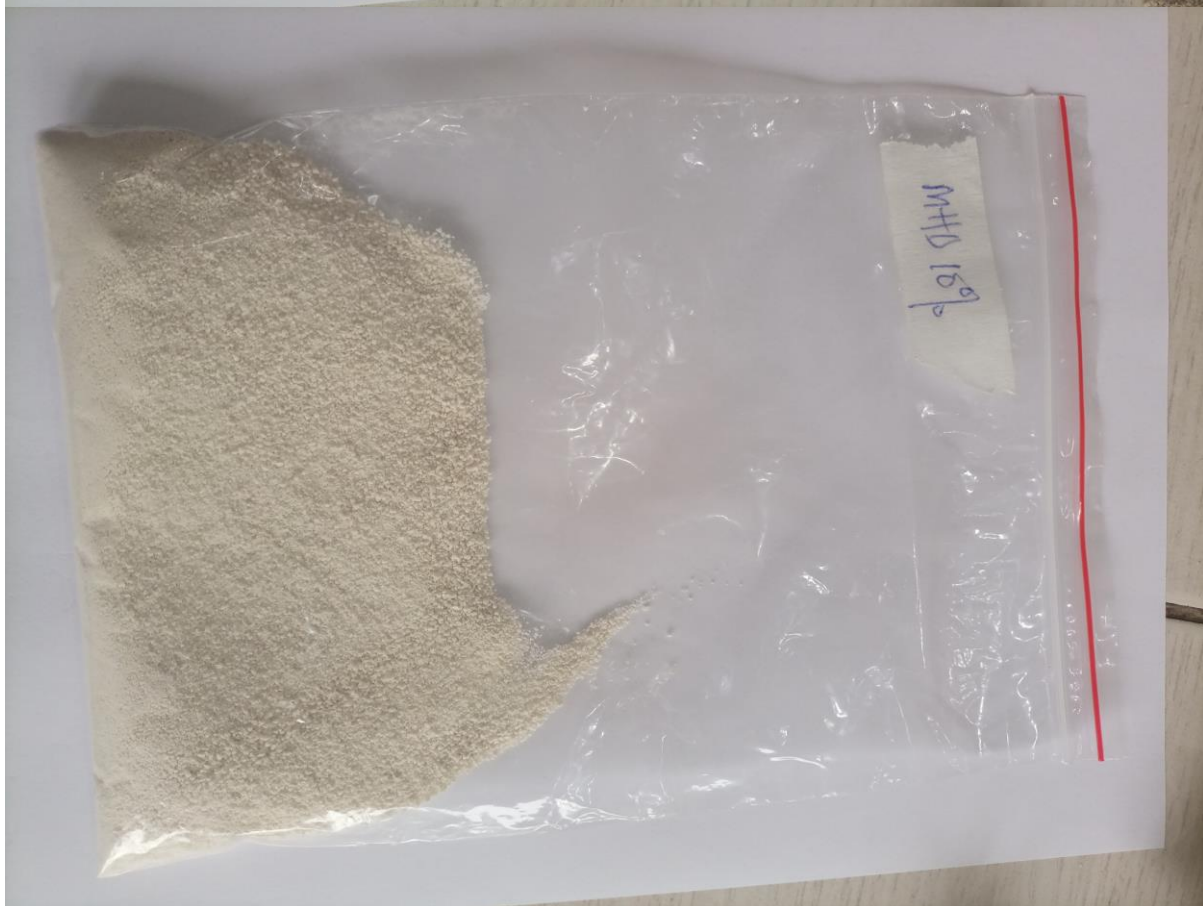

MGD 10%

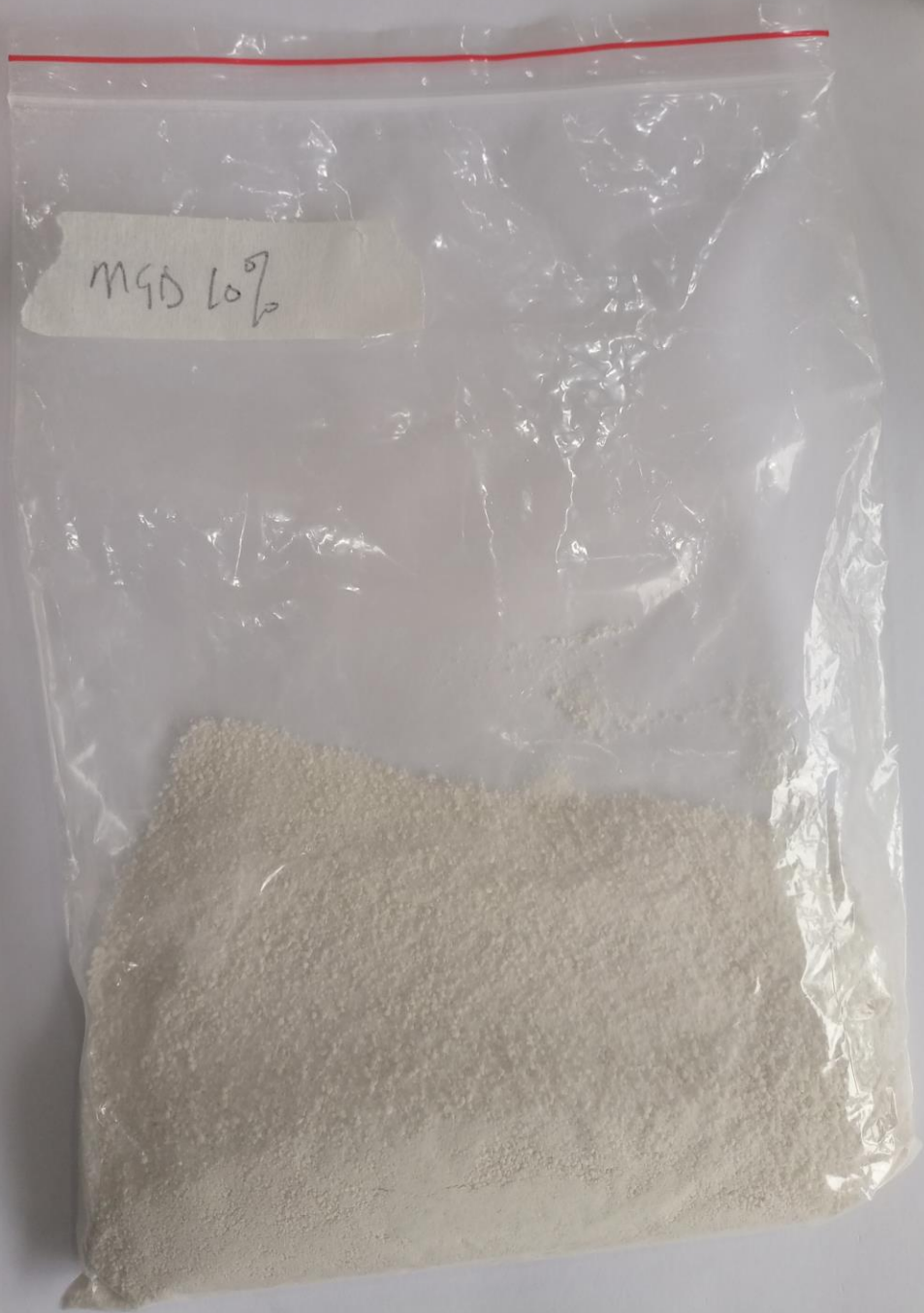

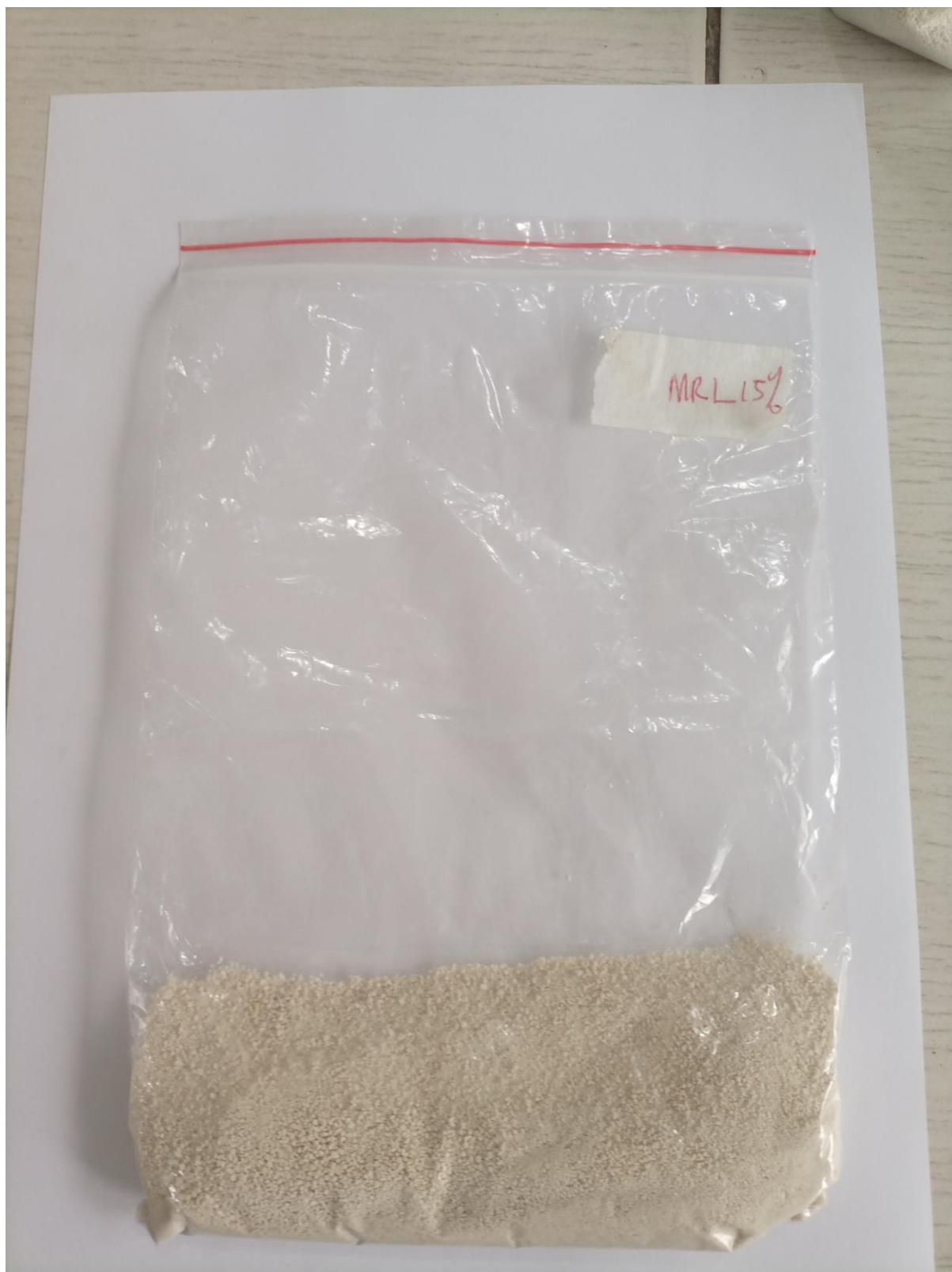

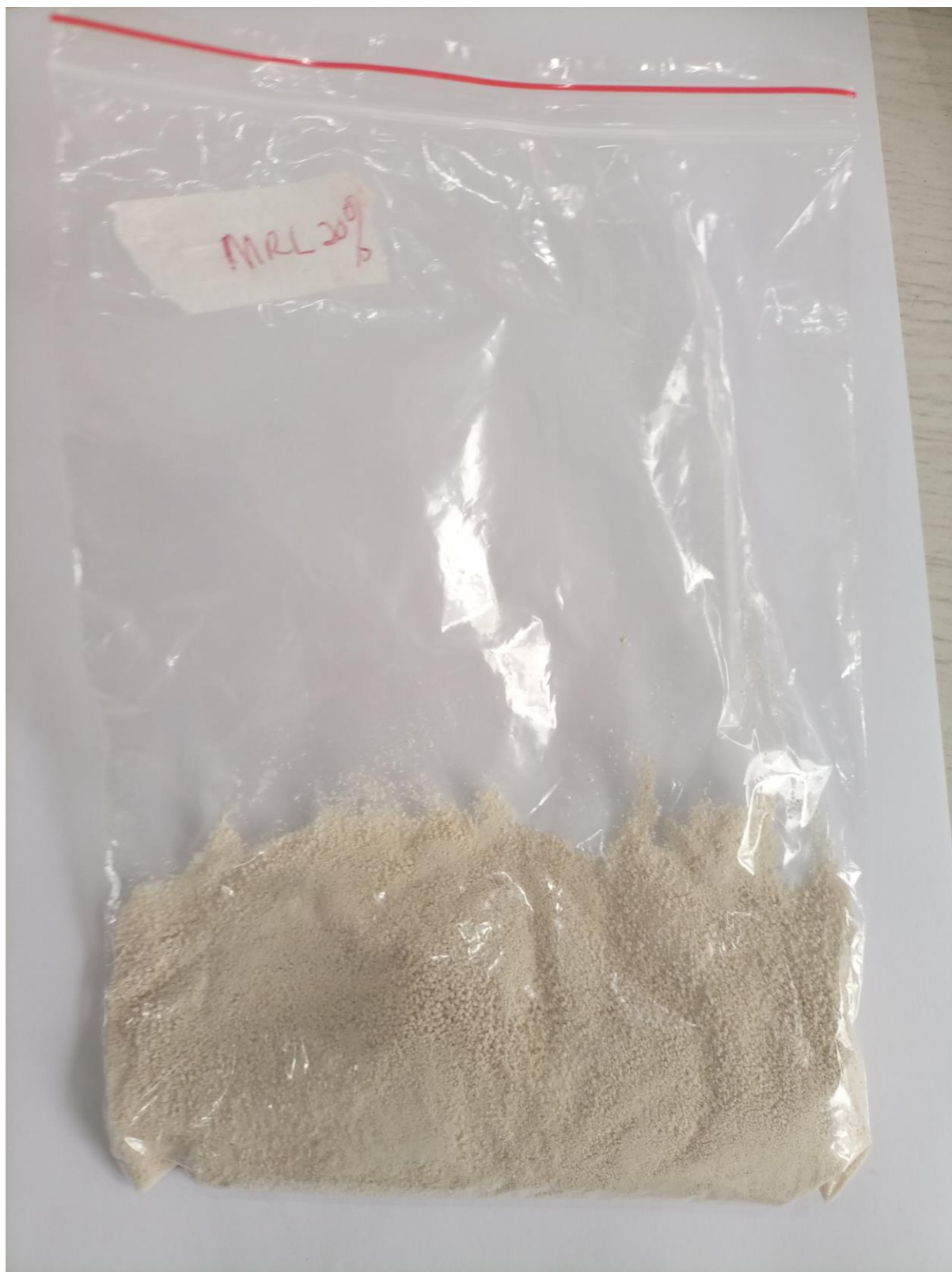

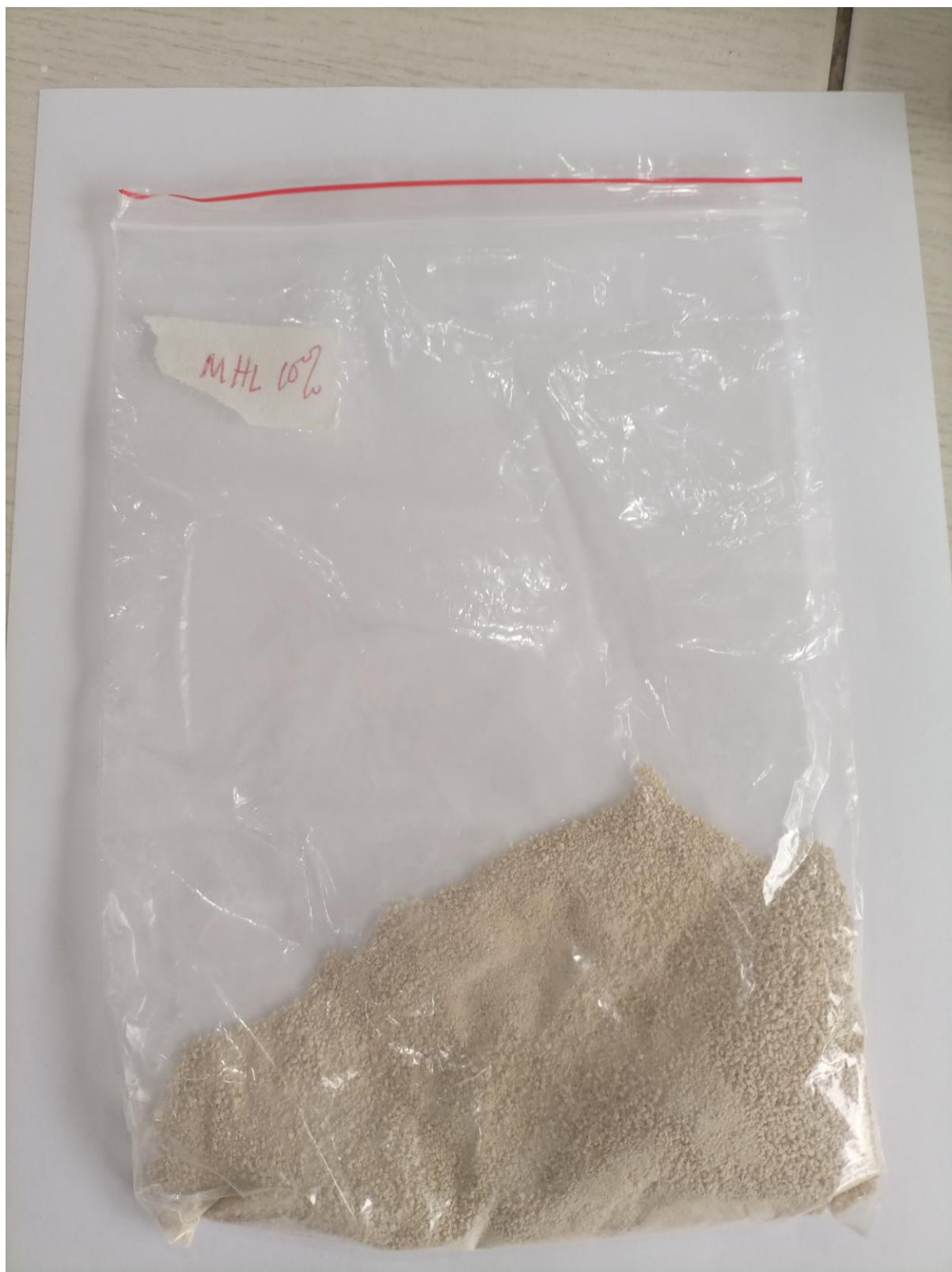

MHL 25%

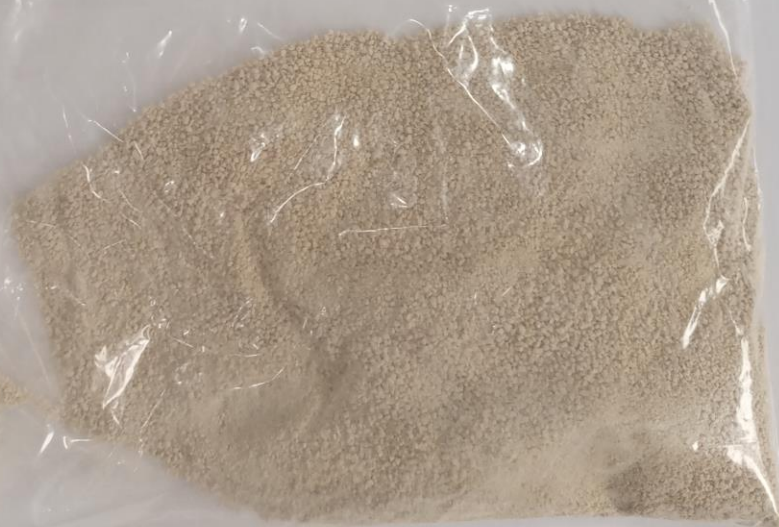

MHL 25%

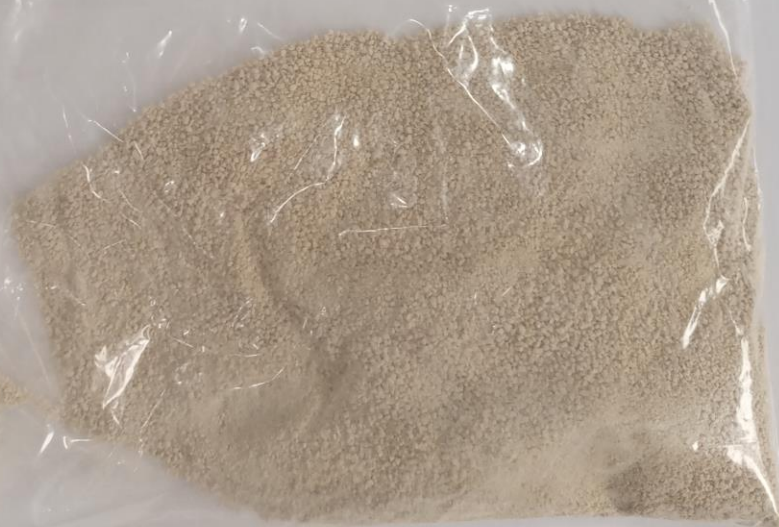

MRL 10%

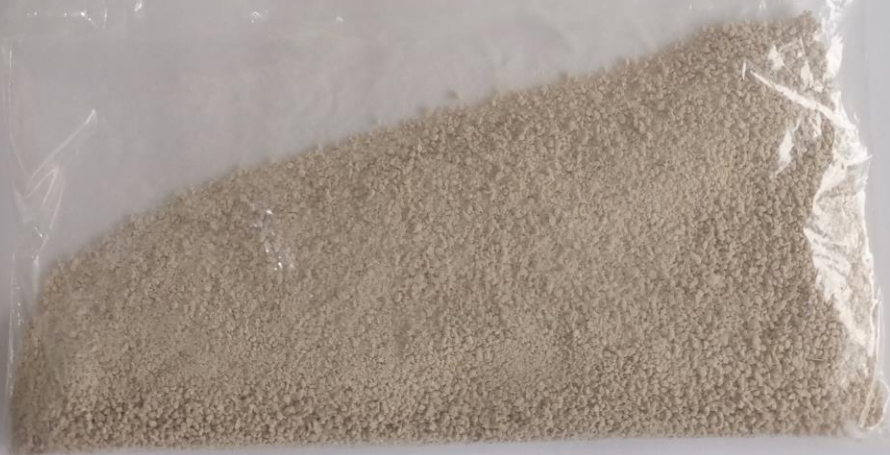

Supplement: Supplementary Materials — Supplementary 1. PPCD: Pre- and postcompression analysis of the formulated tablets. Supplementary 2. FTIR Supplementary: Drug-excipient compatibility IR of the PPP varieties and paracetamol. [file 5461358.f1.zip › PPP Binder Granules.pdf]
